# Impact of water deficiency on leaf cuticle lipids and gene expression networks in cotton (*Gossypium hirsutum* L.)

**DOI:** 10.1186/s12870-022-03788-2

**Published:** 2022-08-17

**Authors:** Fan Yang, Yongchao Han, Qian-Hao Zhu, Xinyu Zhang, Fei Xue, Yanjun Li, Honghai Luo, Jianghong Qin, Jie Sun, Feng Liu

**Affiliations:** 1 Key Laboratory of Oasis Eco-Agriculture, College of Agriculture, Shihezi University, Shihezi, 832000 Xinjiang China; 2 CSIRO Agriculture and Food, GPO Box 1700, Canberra, 2601 Australia; 3 Shihezi Academy of Agricultural Sciences, Shihezi, 832000 China

**Keywords:** *Gossypium hirsutum*, Wax components, Cutin monomers, Transcriptomic analysis, Differentially expressed genes, Co-expression gene network

## Abstract

**Background:**

Water deficit (WD) has serious effect on the productivity of crops. Formation of cuticular layer with increased content of wax and cutin on leaf surfaces is closely related to drought tolerance. Identification of drought tolerance associated wax components and cutin monomers and the genes responsible for their biosynthesis is essential for understanding the physiological and genetic mechanisms underlying drought tolerance and improving crop drought resistance.

**Result:**

In this study, we conducted comparative phenotypic and transcriptomic analyses of two *Gossypium hirsutum* varieties that are tolerant (XL22) or sensitive (XL17) to drought stress. XL17 consumed more water than XL22, particularly under the WD conditions. WD significantly induced accumulation of most major wax components (C29 and C31 alkanes) and cutin monomers (palmitic acid and stearic acid) in leaves of both XL22 and XL17, although accumulation of the major cutin monomers, i.e., polyunsaturated linolenic acid (C18:3n-3) and linoleic acid (C18:2n-6), were significantly repressed by WD in both XL22 and XL17. According to the results of transcriptome analysis, although many genes and their related pathways were commonly induced or repressed by WD in both XL22 and XL17, WD-induced differentially expressed genes specific to XL22 or XL17 were also evident. Among the genes that were commonly induced by WD were the *GhCER1* genes involved in biosynthesis of alkanes, consistent with the observation of enhanced accumulation of alkanes in cotton leaves under the WD conditions. Interestingly, under the WD conditions, several *GhCYP86* genes, which encode enzymes catalyzing the omega-hydroxylation of fatty acids and were identified to be the hub genes of one of the co-expression gene modules, showed a different expression pattern between XL22 and XL17 that was in agreement with the WD-induced changes of the content of hydroxyacids or fatty alcohols in these two varieties.

**Conclusion:**

The results contribute to our comprehending the physiological and genetic mechanisms underlying drought tolerance and provide possible solutions for the difference of drought resistance of different cotton varieties.

**Supplementary Information:**

The online version contains supplementary material available at 10.1186/s12870-022-03788-2.

## Background

Cuticular waxes and cutin are the major structural constituents in the cuticle, which is a hydrophobic lipid covering the epidermal cells layer [1]. As an important strategy of plants to adapt to environmental stresses, cuticular waxes and cutin play a key role in drought tolerance by hindering the appearance of cellular dehydration during drought stress [2, 3]. Cuticle wax is complicated mixtures of long chain fatty acids and their derivatives and its components generally include alkanes, aldehydes, fatty alcohols, ketones, wax esters, etc. [4, 5]. Cutin is embedded in or covered with wax and is comprised of C_16_ and C_18_ fatty acids and corresponding oxygenated derivatives, e.g. hydroxy-fatty acids and dicarboxylic acids [6–9].

Recent studies have established that plants could respond to WD by increasing cuticle wax deposition [1, 10, 11]. It has been reported that tobacco, sesame and soybean gained more wax amount on leaves after short period water shortage [12–14]. WD also resulted in significant increase in the amount of cutin monomers, largely manifested as a high increase in C_16_ and C_18_ dioic acids [1]. Some genes involved in wax and cutin biosynthesis pathway were significantly induced upon drought stress. *ECERIFERUM 1* (*CER1*) encodes aldehyde decarboxylase, which is in charge of converting aldehydes to alkanes [15, 16]. Under low humidity conditions, *Arabidopsis cer1* mutant showed male sterility, while plants with *CER1* overexpression exhibited the decreased cuticle permeability and slower response to soil WD [16]. *Arabidopsis CER3* (*WAX2*) is also involved in wax biosynthesis [17] and *cer3* mutant showed alterations in wax synthesis [18]. Similarly, the mutants defective in one or more cutin monomers, such as *fdh*, *lacs2*, *hth*, *lcr*, and *att1*, showed significant alteration in cutin deposition and cuticle permeability [8, 9, 19–22], revealing important roles of cutin in the formation of water permeability barrier of the cuticle membrane [1]. However, so far, the function of definite cutin monomers to permeability of plant cuticle is still unclear and the role of wax components and cutin monomers and drought responsive genes networks still needs to be further studied.

Cotton is not only the most important textile fiber crop, but also an important oil crop, which has important economic value [23, 24]. WD and drought stress seriously affect cotton yield and quality [25]. It has been reported that WD reduced stomatal conductance, photosynthetic rate, and transpiration rate, and could restrain accumulation of dry matter of cotton plants up to 50% [26]. Regarding the molecular mechanism of drought resistance of cotton, the mitogen-activated protein kinase (MAPK) pathway and calcium signaling pathway have been implicated in drought stress response [25]. Two *MAPK* genes, *GhMPK2* and *GhMPK16* [27, 28], a bZIP transcription factor *GhABF2*, and a R2R3-type MYB transcription factor *GbMYB5* [29, 30] have been demonstrated to be involved in drought stress response. However, few reports have touched on the relationship between the wax components and cutin monomers of cotton leaves and drought stress responses, and little is known about the regulatory metabolic networks involved in drought stress response in cotton.

In this study, we conducted phenotypic and physiological observations on two cotton varieties with different level of drought tolerance under well-watered (WW) and WD conditions, and performed comparative transcriptome analysis using leaf samples collected from WW or WD cotton plants at two developmental stages. The major aims of the study were to uncover the profiles of wax components and cutin monomers of cotton varieties tolerant or sensitive to drought stress under the WW and WD conditions, to identify the major genes and their associated gene networks responding to drought stress, and to understand the potential molecular mechanisms underlying drought tolerance in cotton.

## Materials and methods

### Plant materials and water treatments

Two Upland cotton (*G. hirsutum* L.) varieties (XL17 and XL22, susceptible and tolerant to drought stress, respectively) were legally obtained from Cotton Research Institute, Shihezi University and were used as experimental materials. XL22 (drought tolerant) and XL17 (drought sensitive) were screened through drought tolerance experiment in natural population in our previous study. Compared to that of their respective control plants under the WW conditions, the yield of XL22 decreased by 34.2%, while the yield of XL17 decreased by 41.3% (Table S1). To maintain treatment consistency and comparability, the experiments were carried out in an artificial climate chamber at 28–30 °C, a 16L:8D photoperiod, and 40% relative humidity. The pot experiment was carried out in greenhouse, and each pot (16 cm diameter and 13.7 cm height) was filled with cultivation substrate purchased from the Floragard Vertriebs (Germany).

Two treatments with soil water content of 70% (representing WW) or 10% (representing WD) were used to investigate drought response of cotton plants. Soil moisture was controlled by the weight measurement method according to Hanson [31]. The treatment was adopted after seedling emergence and lasted from seedling stage to bud stage, and each treatment was set up with three replications.

### Leaf area, the relative water content, and stomatal density of leaves

Leaf area of cotton plants was measured according to the method reported by Drake et al. [32] using the inverse fourth leaf of each cotton plant using LI 3100C Area Meter (Li-Cor Inc., USA). The relative water content (RWC) of leaves was determined according to Lu et al. [33]. The stomatal density was measured according to Dunn et al. [34] using a 1 mm^2^ leaf disc sampled from the fourth leaf. The stomatal density was expressed as the number of pores per unit area (mm^2^). The stomatal aperture (µm) was calculated using ImageJ (v1.49) from images collected at × 150 magnification. The average of 12 visual fields of each sample was taken as the result of the sample and every experiment was repeated five times.

### Cuticular wax and cutin analysis

The epicuticular wax of leaf was examined using a scanning electron microscope (SEM) as described by Djanaguiraman et al. [35]. The total epicuticular wax content was measured using chloroform extract from cotton leaves according to a previously reported method [33]. The composition and amount of wax were analyzed using a published protocol [36]. The constituent analyses were performed using GCMS-QP2020 with a DB-1 column of 30 m × 0.32 mm and film thickness of 0.1 μm. GC–MS analyses were performed as described by Liu et al. [37]. Each compound was quantified against the internal standard by automatic integration of the peak areas.

The analyses of total cutin content and monomer were performed according to Franke et al. [38] with some modifications. The extraction was performed at room temperature in glass tubes with teflon-lined screw caps. Soluble lipids were removed by dipping the cotton leaf in 10 mL of a methanol:chloroform (1:1, v/v) mixture with continuous agitation for 7 days and the solvent was changed daily. The samples were dried under a gentle stream of nitrogen gas and then the leaf lipid polyesters were depolymerizated in 6 ml methanol/sulfuric acid/chloroform (10:0.5:1/, v/v/v) mixture at 80 °C with occasional agitation. The resulting cutin monomer fraction was derivatized with BFTSA/pyridine (1:1) for 60 min at 70 °C. Samples were analyzed using GCMS-QP2020 according to Liu et al. [37] and each compound was quantified on the basis of its total ion current as described by Li-Beisson et al. [36].

### RNA isolation, library preparation and sequencing

The extraction, purification and integrity identification of total RNA were carried out according to our previously published methods [24, 37]. The libraries were sequenced on an Illumina Hi-seq platform. Raw reads were firstly processed through in-house perl scripts [39]. And then, clean reads were obtained by removing low quality reads and reads containing adapter or ploy-N. Using Hisat2 v2.0.5, paired-end clean reads were aligned to the TM-1 reference genome [40]. FeatureCounts v1.5.0-p3 was used to count the read numbers mapped to each gene. Fragments per kilobase of transcript per million mapped reads (FPKM) of each gene was calculated based on the length of the gene and the number of reads mapped to the gene.

### Differential expression, gene function annotation, enrichment analysis and co-expression networks of differentially expressed genes (DEGs)

Differential expression analysis was performed using the DESeq R package (1.20.0). The resulting *p-value* was adjusted for controlling the false discovery rate according to Benjamini and Hochberg [41]. The adjusted *p-value* < 0.05 and |log_2_ (fold change) |≥ 2 were used as the criteria for identification of DEGs. Gene Ontology (GO) enrichment analysis of DEGs was implemented by the clusterProfiler R package, in which gene length bias was corrected. GO terms with a corrected *p-value* less than 0.05 were considered to be significantly enriched. The clusterProfiler R package was used to test the statistical enrichment of DEGs in KEGG pathways. The co-expression networks among DEGs was constructed by weighted correlation network analysis (WGCNA) software package according to Ma et al. [24] and Cheng et al. [42]. The hub gene was screened and correlation networks were drawn according to Ma et al. [24] and Cheng et al. [42].

### Quantitative RT-PCR validation of DEGs

Gene specific primers were designed using cDNA sequences of the target genes with Primer Premier program (Table S2). qRT-PCR was carried out as described by Cheng et al. [42]. Three independent biological experiments were performed for each sample of each time point. The relative expression levels were calculated with the cotton ubiquitin gene (*GhUBI*, XM_012634824) as the reference according to Cheng et al. [42].

## Results

### Effects of WD on physiological and morphological characteristics of cotton varieties with different level of drought tolerance

Under the WW conditions, i.e., when the soil moisture was kept at 70% of its water holding capacity, the drought tolerant variety XL22 consumed 190 g water per day from the seedling stage (30 days after emergence) to the bud stage (50 days after emergence), and the drought sensitive variety XL17 consumed 206.7 g water per day during the same time period. Under the WD conditions, i.e., when the soil water content was kept at 10% of its water holding capacity, from the seedling stage to the bud stage, XL22 and XL17 consumed 60 g and 90 g water per day respectively (Fig. S1). It thus seems that the drought tolerant variety (XL22) consumed less water than the drought sensitive variety XL17 under both the WW and WD conditions.

**Fig. 1:**
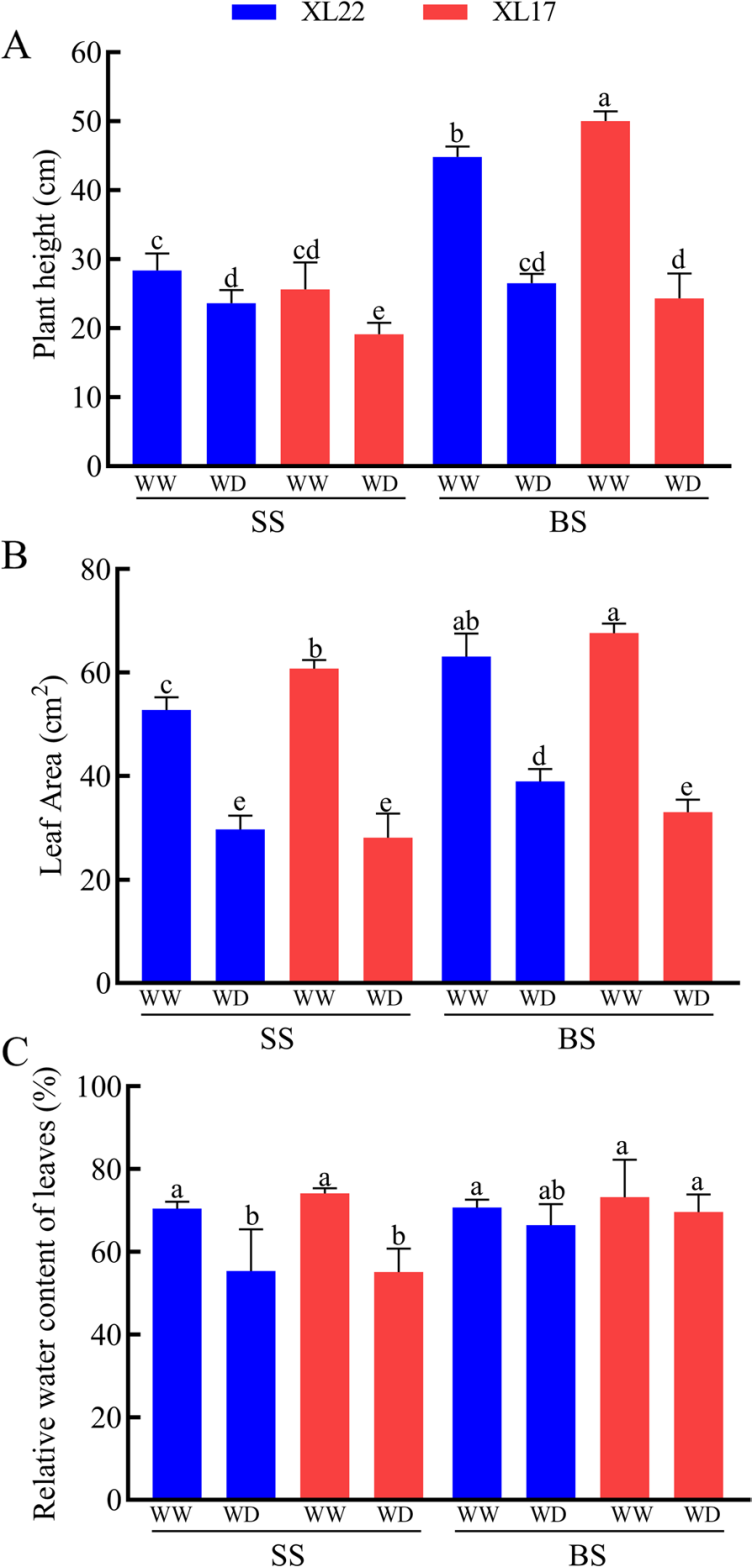
The effect of WD on the physiological and morphological characteristics of cotton variety tolerant (XL22) or sensitive (XL17) to drought. **A** Comparison of plant height. **B** Comparison of leaf area. **C** the relative water content of leaves. SS, seedling stage; BS, bud stage. WW, well-watered; WD, water deficit. Error bars are standard errors. Values represent the means ± SE, *n* = 3. Different letters above the bars indicate statistically different from each other as determined by the Student’s *t* test: *p* < 0.05

Compared to their respective control plants under the WW conditions, both XL17 and XL22 decreased significantly in plant height under the WD conditions at the seedling and the bud stages, particularly at the bud stage, due to a more significant effect of WD on the growth of the drought sensitive variety (XL17) than that of the drought tolerant variety (XL22) (Fig. 1A). From the seedling stage to the bud stage, the plant height of XL22 increased by 57.7 and 12.3% under the WW and WD conditions, respectively. During the same period, the plant height of XL17 increased by 95.3 and 27.2% under the WW and WD conditions, respectively. The results suggested that the effect of WD on plant height growth of XL22 was 45.4%, and that of XL17 was 68.1%. WD reduced the leaf area of XL22 by 43.6 and 38.2% at the seedling stage and the bud stage, respectively. The leaf area of XL17 seemed to be more inhibited by WD, with a decrease of 53.7% and 51.1% observed at the seedling and bud stages, respectively (Fig. 1B). In both XL22 and XL17, the RWC (relative water content) of leaves was always higher under the WW conditions than under the WD conditions. After the WD treatment, the RWC of XL22 leaves was reduced by 15.51% from the seedling stage to the bud stage, while that of XL17 leaves was decreased by 20.8% (Fig. 1C). These results showed that XL17 was more sensitive to WD than XL22, likely due to its weaker ability in maintaining the RWC of plants.

**Fig. 2:**
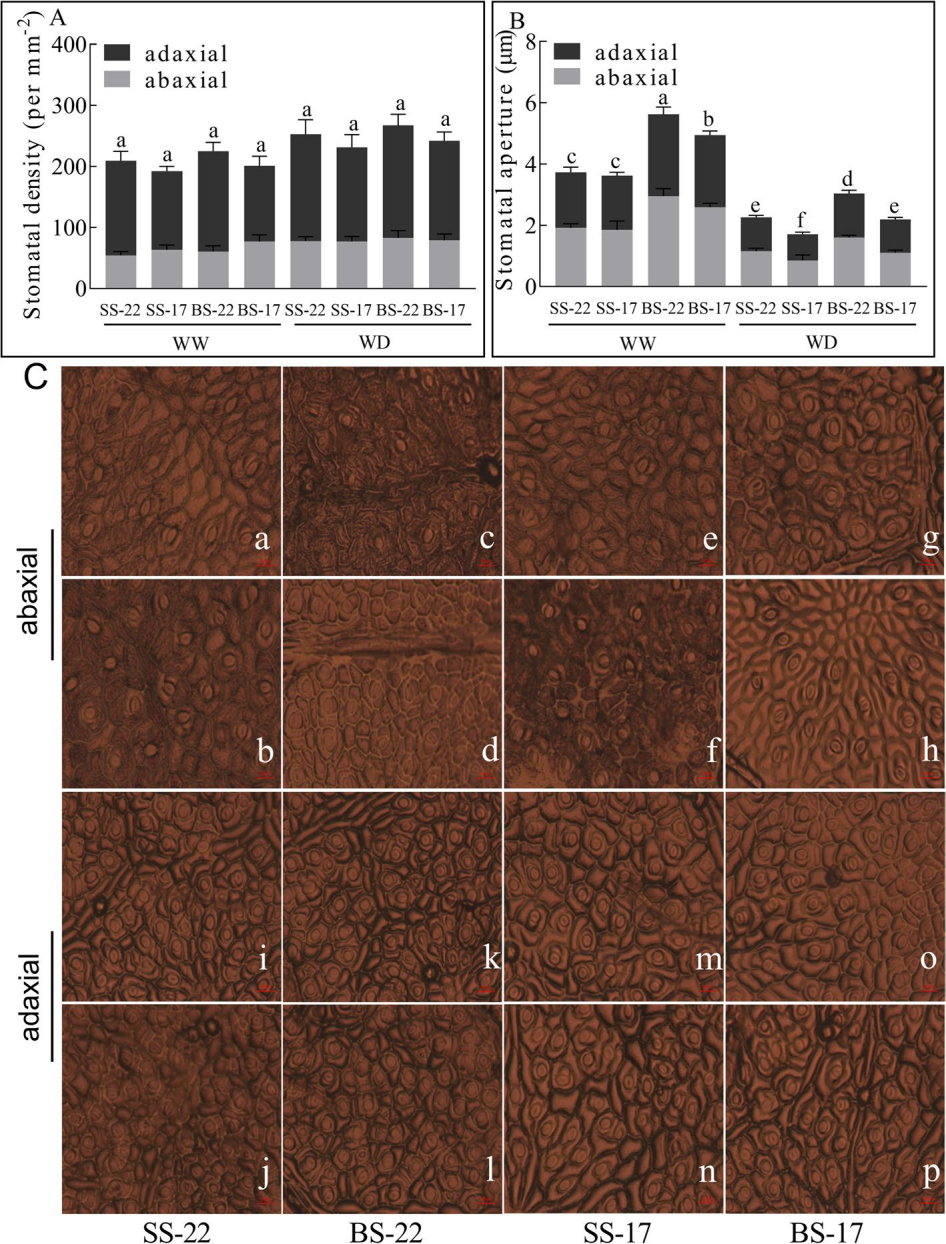
Comparison of the leaf stomatal density and stomatal aperture of cotton variety tolerant (XL22) or sensitive (XL17) to drought under the WW andWD conditions. **A** Leaf stomatal density of the two cotton varieties **B** Leaf stomatal aperture of the two cotton varieties. **C** Leaf impressions showing leaf stomatal apertere at two stages under WW and WD conditions on abaxial and adaxial surfaces. **a** and **b**. XL22 leaf at the seedling stage under WW and WD condition, respectively. **c** and **d**. XL22 leaf at the bud stage under WW and WD condition, respectively. **e** and **f**. XL17 leaf at the seedling stage under WW and WD condition, respectively. **g** and **h**. XL17 leaf at the bud stage under WW and WD condition, respectively. **i**, **j**, **k** and **l**, the adaxial surface of XL22 leaf corresponds to **a**, **b**, **c** and **d**. **m**, **n**, **o** and **p**, the adaxial surface of XL17 leaf corresponds to **e**, **f**, **g** and **h**. SS-22, XL22 leaves at seedling stage; SS-17, XL17 leaves at seedling stage; BS-22, XL22 leaves at bud stage; BS-17, XL17 leaves at bud stage. Error bars are standard errors. Values represent the means ± SE, *n* = 3. The data in Fig. 2A and B were analyzed by analysis of variance (ANOVA) and mean comparison between treatments was performed based on Duncan's multiple range method at 5% level. For Fig. 2A and B, different letters above the bars indicate statistically different (*p* < 0.05)

Leaf stomatal density is a primary determinant of water use efficiency. The adaxial side of leaves had higher stomatal density than that of abaxial side for XL22 and XL17 (Fig. 2A). No significant difference in leaf stomatal density per unit leaf area was observed between XL22 and XL17 under the WW conditions. At the seedling stage, the stomatal density of XL17 and XL22 on the abaxial side increased by 22.55 and 43.18% under the WD conditions, respectively, compared to that of their respective control plants under the WW conditions. At the bud stage, the abaxial stomatal density of XL22 increased by 36.73% under the WD conditions, compared to that of XL22 under the WW conditions. However, WD increased only 2.81% (statistically insignificant) of the abaxial stomatal density in XL17 at the bud stage. Under the WD conditions, the stomatal density on the adaxial side also increased, although the relative proportion of increase was not as much as that of on the abaxial side. The highest stomatal density was observed on adaxial side in the bud stage of XL22 under the WD conditions, with an average density of 184 stomata per mm^2^ (Fig. 2A). WD-induced increase in stomatal density on both leaf surfaces was accompanied by decrease of stomatal aperture in both XL17 and XL22 of the two developmental stages, particularly at the bud stage. For instance, compared to the WW plants, the WD plants of XL22 and XL17 showed a 45.54 and 57.27% decrease of the stomatal aperture, respectively (Fig. 2B). WD-induced changes in stomatal aperture were even more apparent on the adaxial surface than on the abaxial surface (Fig. 2C).

### Effect of WD on cuticle lipids in leaves

**Fig. 3:**
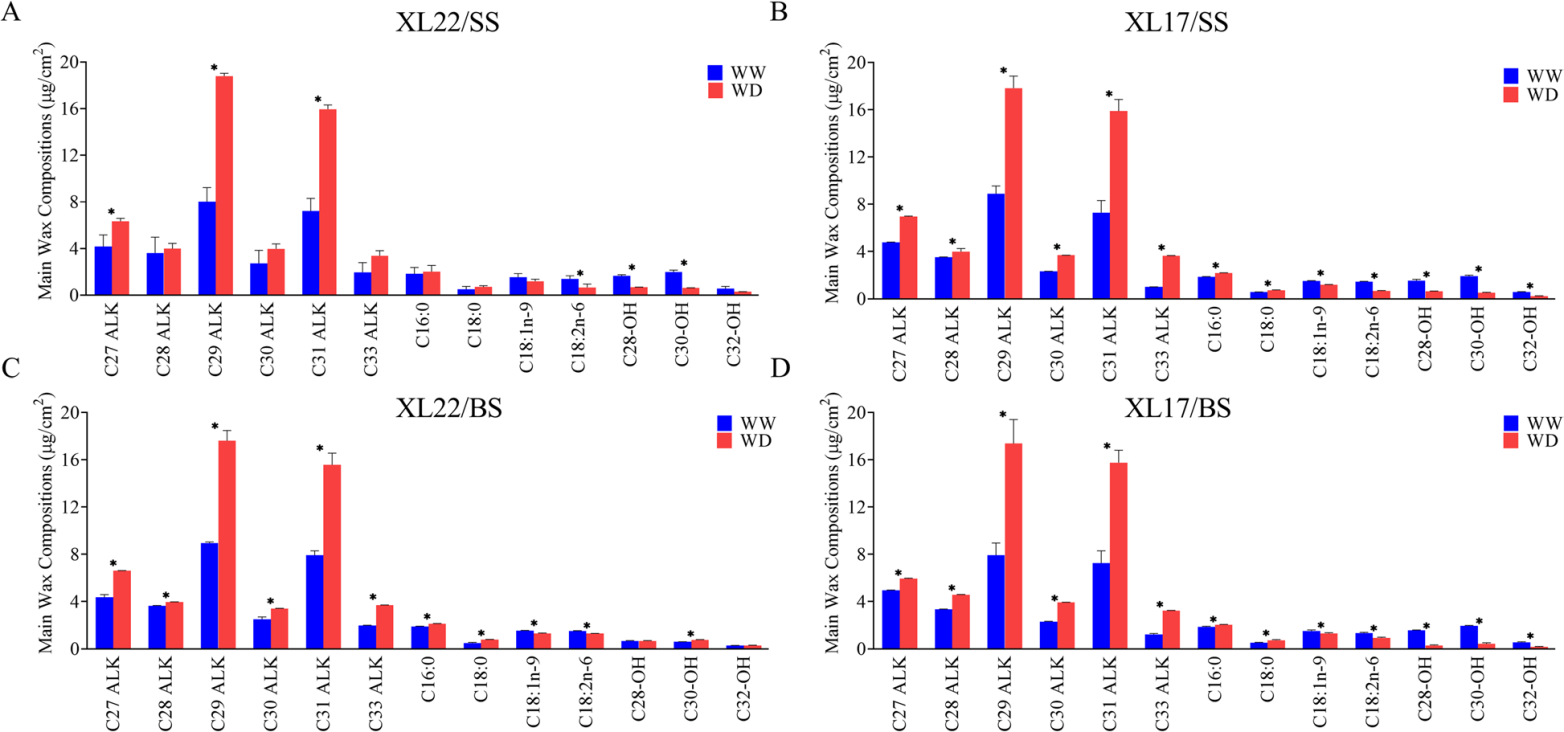
The effect of WD on the accumulation of wax constituents in XL22 and XL17 leaves. **A** and **C** The main wax constituents in XL22 leaves at the seedling stage and bud stage, respectively. **B** and **D** The main wax constituents in XL17 leaves at the seedling stage and bud stage, respectively. C27 ALK, heptacosane; C28 ALK, octacosane; C29 ALK, nonacosane; C30 ALK, triacontane; C31 ALK, hentriacontane; C33 ALK, tritridecane. C16:0, hexadecanoic acid; C18:0, octadecanoic acid; C18:1n-9, 9-octadecenoic acid; C18:2n-6, 9,12-octadecadienoic acid. C28-OH, octacosanol; C30-OH, triacontanol; C32-OH, dotriacontanol. WW, well-watered; WD, water deficit. Error bars are standard errors. Values represent the means ± SE, *n* = 3. Asterisks denote significant difference as determined by the Student’s *t* test: *p < 0.05. XL22/SS, XL22 leaves at seedling stage; XL17/SS, XL17 leaves at seedling stage; XL22/BS, XL22 leaves at bud stage; XL17/BS, XL17 leaves at bud stage

Cuticular wax formation on the surface of plant leaves is thought to be associated with drought stress tolerance. We thus compared changes of cuticular wax and cutin in the leaves of XL22 and XL17 under the WW and WD conditions to explore the stoma-independent drought tolerant mechanisms. Under the WW conditions, no difference in leaf wax content was evident between XL22 and XL17. After the WD treatment, the leaf surface of both XL22 and XL17 appeared, based on visual inspection, to be covered by more wax at both the seedling and bud stages, particularly at the bud stage. Further observation by SEM, there was no significant difference in waxy crystals between XL22 and XL17 (Fig. S2). After WD treatment, the surface of cotton leaves was covered with more wax, and waxy crystals on leaves front side was more apparent (Fig. S2). The observation was also supported by the result of significant increase of total wax amount per unit leaf area in both varieties. Under the WW conditions, the total wax content of XL22 leaves was 40.35 µg/cm^2^ and 40.59 µg/cm^2^ at the seedling stage and bud stage, respectively. The corresponding values in XL17 were 40.16 µg/cm^2^ and 40.38 µg/cm^2^. Under the WD conditions, the total wax content of XL22 leaves was 64.06 µg/cm^2^ and 64.75 µg/cm^2^ at the seedling stage and bud stage, respectively. The corresponding values in XL17 were 63.02 µg/cm^2^ and 63.22 µg/cm^2^. There was no significant difference in the total wax content between XL22 and XL17 leaves under the WD conditions (Fig S3). Leaf wax constituents were mainly divided into alkanes, fatty acids, and primary alcohols (Fig. 3). Alkanes with a carbon chain length ranging from 27 to 33 were the major components of leaf wax constituents and accounted for over 90% increase of the total wax observed in the WD plants, consistent with the result reported in *Arabidopsis* [1]. Of the six alkanes, C29 and C31 ones had a higher basal level than other four and were also the most significantly induced by WD. The contents of fatty acids and primary alcohols were relatively low in leaf wax, but significant decrease was observed for most of their components in leaves of the WD plants (Fig. 3). The major primary alcohols are octacosanol (C28-OH) and triacontanol (C30-OH) in the total wax content of cotton leaves. Under the WW conditions, the total content of primary alcohols was respectively 4.18 µg/cm^2^ and 4.04 µg/cm^2^ at the seedling stage of XL22 and XL17. Under the WD conditions, its content significantly decreased by 62.1% for XL22 and 66.5% for XL17, respectively. The reduction of primary alcohols at the bud stage seemed to be more significant in XL17 than in XL22 under the WD conditions. Under the WD conditions, the total content of primary alcohols was respectively 1.73 µg/cm^2^ and 1.02 µg/cm^2^ at the bud stage of XL22 and XL17 (Fig. 3). Generally, XL22 and XL17 had a very similar profile of wax components under both the WW and WD conditions, but reduction of primary alcohols at the bud stage seemed to be more significant in XL17 than in XL22 under the WD conditions.

**Fig. 4:**
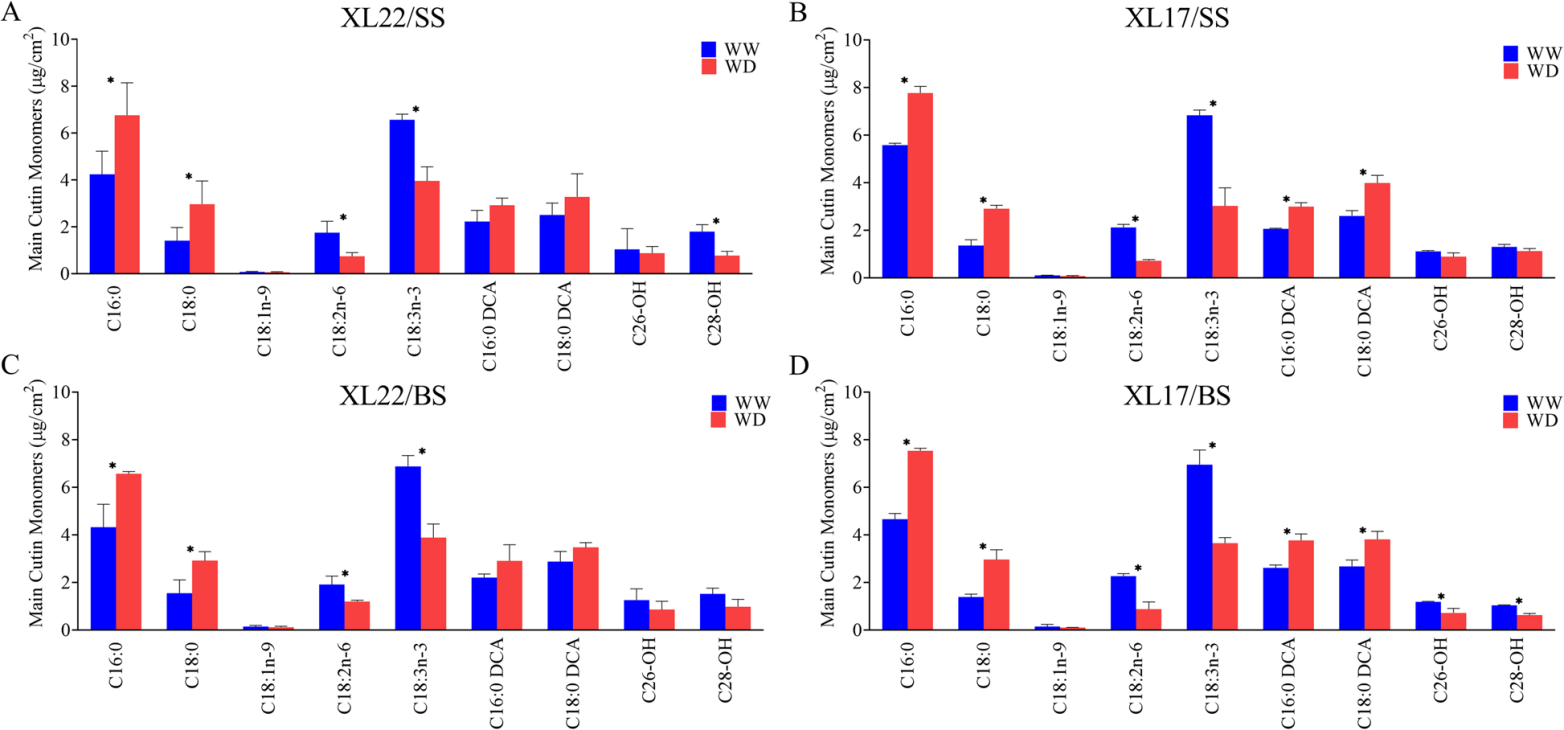
The effect of WD on the accumulation of cutin monomers in XL22 and XL17 leaves. **A** and **C** The main cutin monomers in XL22 leaves at the seedling stage and bud stage, respectively. **B** and **D** The main cutin monomers in XL17 leaves at the seedling stage and bud stage, respectively. C16:0, hexadecanoic acid; C18:0, octadecanoic acid; C18:1n-9, 9-octadecenoic acid; C18:2n-6, 9,12-octadecadienoic acid. C18:3n-3, 9,12,15-octadecatrienoic acid. C16:0 DCA, hexadecane-1,16-dioic acid; C18:0 DCA, octadecanedioic acid. C26-OH, hexacosanol; C28-OH, octacosanol. WW, well-watered; WD, water deficit. Error bars are standard errors. Values represent the means ± SE, *n* = 3. Asterisks denote significant difference as determined by the Student’s *t* test: *p < 0.05. XL22/SS, XL22 leaves at seedling stage; XL17/SS, XL17 leaves at seedling stage; XL22/BS, XL22 leaves at bud stage; XL17/BS, XL17 leaves at bud stage

According to the GC–MS results (Fig. 4), the main cutin monomers identified in cotton leaves included linolenic acid (C18:3n-3), palmitic acid (C16:0), linoleic acid (C18:2n-6), stearic acid (C18:0), oleic acid (C18:1n-9), hexadecane-1,16-dioic acid (C16:0 DCA), and octadecanedioic acid (C18:0 DCA). Hexacosanol (C26-OH) and octacosanol (C28-OH) were also detected in cotton leaves (Fig. 4). The most abundant cutin monomer in cotton leaves was C18:3n-3, accounting for more than 20% of the total cutin content. Similar to the wax profile, the cutin profile was generally similar between XL22 and XL17 at both developmental stages, for instance, the content of polyunsaturated fatty acids, C18:2n-6 and C18:3n-3, decreased significantly in both varieties in the WD plants (Fig. 4). But the WD-induced increase of C16:0 DCA and C18:0 DCA was significant only in drought sensitive XL17 but not in drought tolerant XL22. In addition, although WD-induced reduction of hexacosanol (C26-OH) was observed in both XL22 and XL17 at both developmental stages, a significant reduction of C26-OH was observed only in the drought sensitive XL17 at the bud stage (Fig. 4).

### Transcriptome analysis

To explore the molecular mechanism underlying drought stress response and the difference of drought tolerance between XL22 and XL17, we compared leaf transcriptomes of XL22 and XL17 at the seedling stage and the bud stage. In total, 24 libraries (three biological repetitions for the two varieties under each treatment) were sequenced and a total of 1,116,540,102 raw reads were generated. The average Q30 of the reads was 94.48% and the read GC content was 44.20%. Raw reads were filtered to remove low quality ones and a total of 1,092,594,914 clean reads were finally used in alignment. Approximately 97.86% of the clean reads could be aligned to the TM-1 reference genome with ~ 93.08% of them being uniquely aligned (Table S3). These results suggested that the RNA-seq data were high quality and suitable for further analyses.

**Fig. 5:**
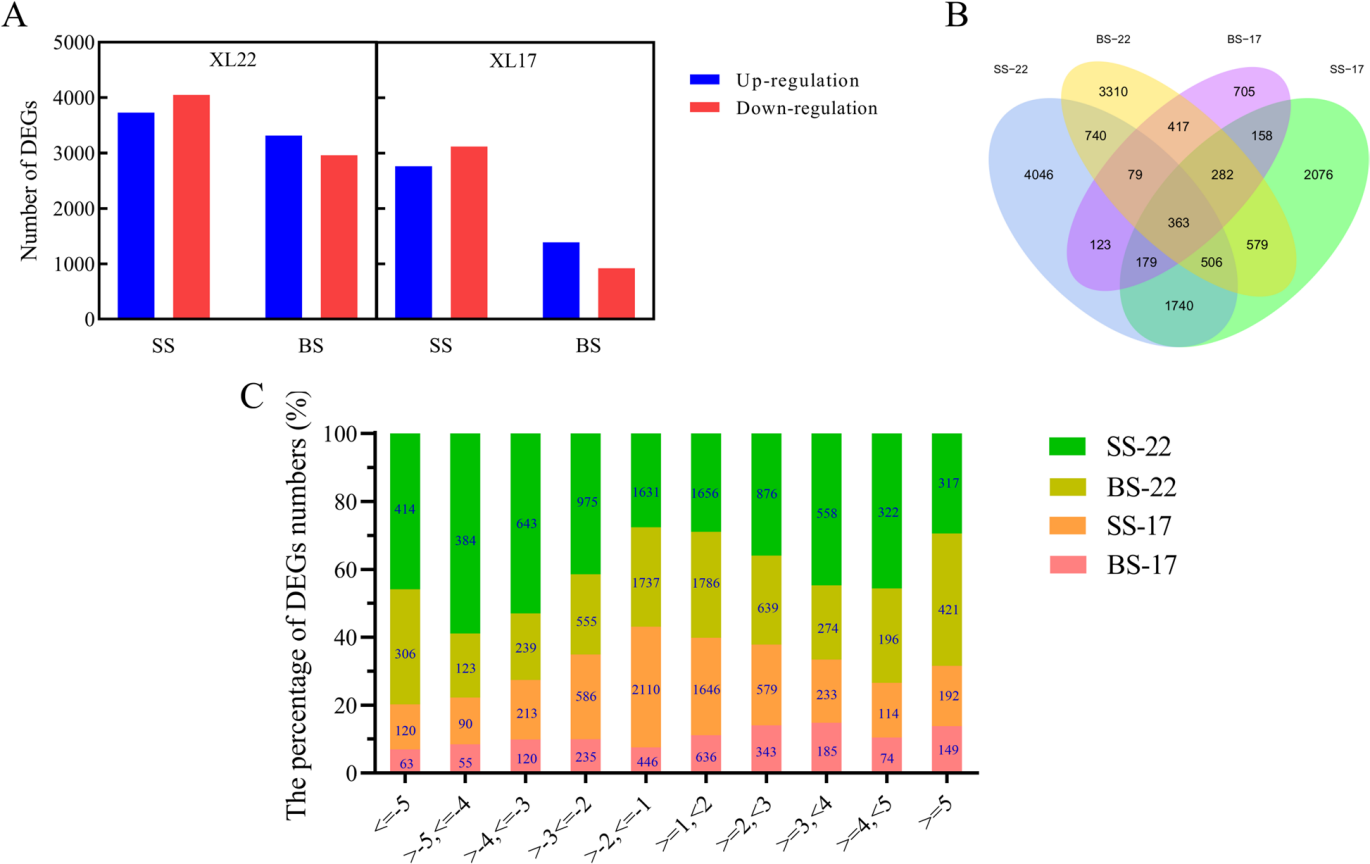
The number and distribution of DEGs under the WW and WD conditions. **A** The number of the up-regulated or down-regulated DEGs (WD *vs* WW) in XL22 and XL17. **B** Venn diagram showing overlapping and unique DEGs in different varieties and developmental stages. **C** The distribution of DEGs with different fold changes. SS-22, XL22 leaves at seedling stage; SS-17, XL17 leaves at seedling stage; BS-22, XL22 leaves at bud stage; BS-17, XL17 leaves at bud stage

Using the criteria mentioned in Methods, we identified a total of 22,241 genes to be differentially expressed between the WW and WD plants of XL22 or XL17. Of those differentially expressed genes (DEGs), 11,196 (50.34%) were up-regulated (WD *vs* WW) and 11,045 (49.66%) were down-regulated (*P*_*adj*_ < 0.05; Fig. 5). In both XL22 and XL17, there were more DEGs at the seedling stage than at the bud stage. For XL22, compared with the WW treatment, the WD treatment resulted in a slightly higher number of down-regulated genes than that of up-regulated genes at both developmental stages, and for XL17, this was observed only at the seedling stage but not at the bud stage (Fig. 5A). Of the 22,241 DEGs, 15,303 were non-redundant ones, with 4,046 and 3,310 unique to the seedling and bud stage of XL22, respectively, and 2,076 and 705 unique to the seedling and bud stage of XL17, respectively. A total of 363 DEGs were common between XL22 and XL17 at both developmental stages (Fig. 5B). Of the up-regulated genes in both varieties, 2,437 (21.77%) had a 2–3 folds change in expression while 1,079 (9.64%) had an over 5 folds change in gene expression. Among the down-regulated genes, 2,351 (21.29%) had a 2–3 folds expression difference while 903 (8.18%) had a > 5 folds expression difference (Fig. 5C).

### DEGs were confirmed by qRT-PCR analysis

To verify the results of the RNA-seq and identify DEGs, the expression levels and expression trend of 9 genes were analyzed by qRT-PCR, including 5 genes related to wax and cutin biosynthesis (Fig. S4). The results of qRT-PCR of the selected genes were highly identical with those of RNA-seq, showing that the results of RNA-seq were highly reliable (Fig. S4).

### Gene ontology analysis of DEGs

**Fig. 6:**
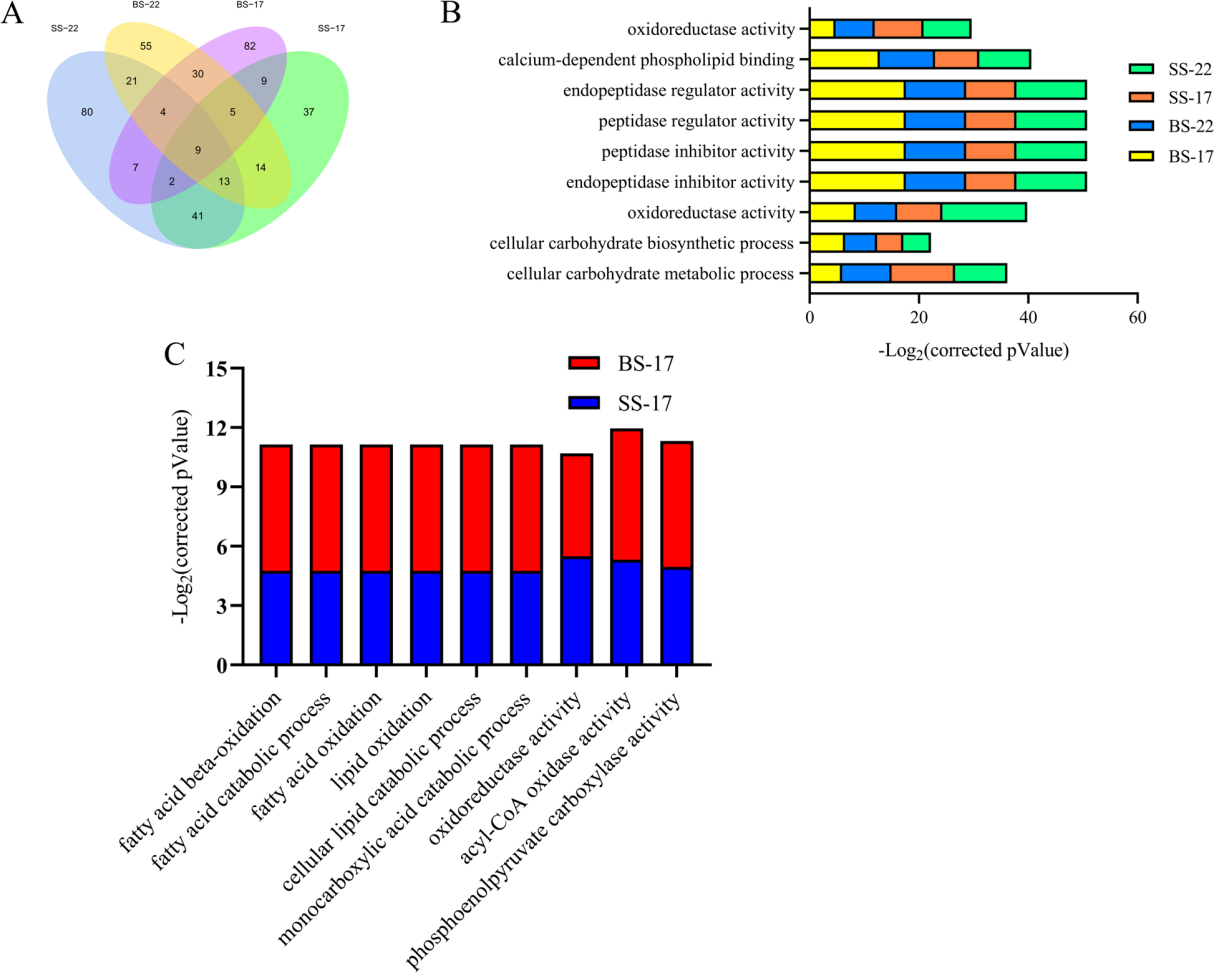
Gene ontology classification of DEGs between the well-watered and water deficit XL22 and XL17 plants. **A** Venn diagram showing the number of enriched GO terms overlapping or specific to each developmental stage of the two cotton varieties. **B** Enriched GO terms at the seedling stage and the bud stage of XL22 and XL17. **C** Enriched GO terms at the seedling stage and the bud stage of XL17. SS-22, the seedling stage of XL22; SS-17, the seedling stage of XL17; BS-22, the bud stage of XL22; BS-17, the bud stage of XL17

Gene Ontology (GO) analysis was performed using the identified DEGs between the WW and WD plants. Using the criterion of corrected *P*_*Value*_ ≤ 0.05, we found that the 15,303 non-redundant DEGs were enriched for 205 GO terms (Table S4). Of the 205 GO terms, 177 (including 80 unique ones) and 151 (including 55 unique ones) were enriched at the seedling and the bud stage of XL22, respectively, and 130 (including 37 unique ones) and 148 (including 82 unique ones) were enriched at the seedling and the bud stage of XL17, respectively (Fig. 6A). Twenty one GO terms were enriched at both the seedling and bud stages of XL22, and 9 GO terms were enriched at both the seedling and bud stages of XL17. Forty one GO terms were enriched at the seedling stage of both XL22 and XL17 and 30 GO terms were enriched at the bud stage of both XL22 and XL17 (Fig. 6A). A total of 9 GO terms were commonly enriched at the seedling and bud stages of both XL22 and XL17 (Fig. 6B). The top enriched GO terms were quite different between XL22 and XL17. The top two biological process terms enriched in XL22 were related to multi-organism process and DNA replication (Table S5), whereas those enriched in XL17 were related to fatty acid beta-oxidation and fatty acid catabolic process (Fig. 6C), suggesting that different biological processes could be responsible for the variable drought response of XL22 and XL17, although certain drought induced biological processes are shared by the two varieties.

### KEGG pathways of DEGs

We further performed Kyoto Encyclopedia of Genes and Genomes (KEGG) pathway analysis according to Kanehisa et al. [43] using the 15,303 non-redundant DEGs and found that the DEGs were associated with 119 pathways (Table S6). Many of the significantly changed pathways were related to alpha-linolenic acid metabolism, cutin and suberine and wax biosynthesis, fatty acid metabolism, starch and sucrose metabolism, peroxisome, steroid biosynthesis, and biosynthesis of unsaturated fatty acids (Table S6). The KEGG pathways enriched at the seedling stage of XL22 included those involved in metabolism of alpha-linolenic acid, linoleic acid, galactose, starch and sucrose, biosynthesis of unsaturated fatty acids, and biosynthesis of steroid, cutin, suberine and wax (Fig. S5A). At the seedling stage of XL17, pathways related to metabolism of starch, sucrose, fatty acids, alpha-linolenic acid, porphyrin, and chlorophyll were enriched (Fig. S5B). *GH_D06G1740* encoding transcription factor bHLH91, *GH_D09G1061* encoding alcohol-forming fatty acyl-CoA reductase, *GH_D11G3608* encoding delta 12-fatty-acid desaturase (*FAD2*), and *GH_A11G3588* involved in the biosynthesis of unsaturated fatty acids were among the DEGs involved in the pathways related to biosynthesis of cutin, suberine and wax (Fig. S6A-Fig. S6C, Table S6). In addition to the aforementioned *GH_A11G3588* and *GH_D11G3608*, *GH_D01G2159* encoding alcohol dehydrogenase class-P, and two genes, *GH_A05G0687* and *GH_D05G0687*, encoding linoleate 13S-lipoxygenase 2–1 were found in the enriched pathways (Fig. S6D-Fig. S6E, Table S6). The pathways enriched at the bud stage of XL22 were related to metabolism of linoleic acid and biosynthesis of steroid, and the related DEGs included *GH_A05G0687* and *GH_D05G0687* that encode linoleate 13S-lipoxygenase 2–1. These two genes seemed to be also involved in the pathways enriched at the bud stage of XL17 that were related to jasmonate/oxylipin biosynthesis.

### Expression profiles of the DEGs involved in wax and cutin biosynthesis

**Fig. 7:**
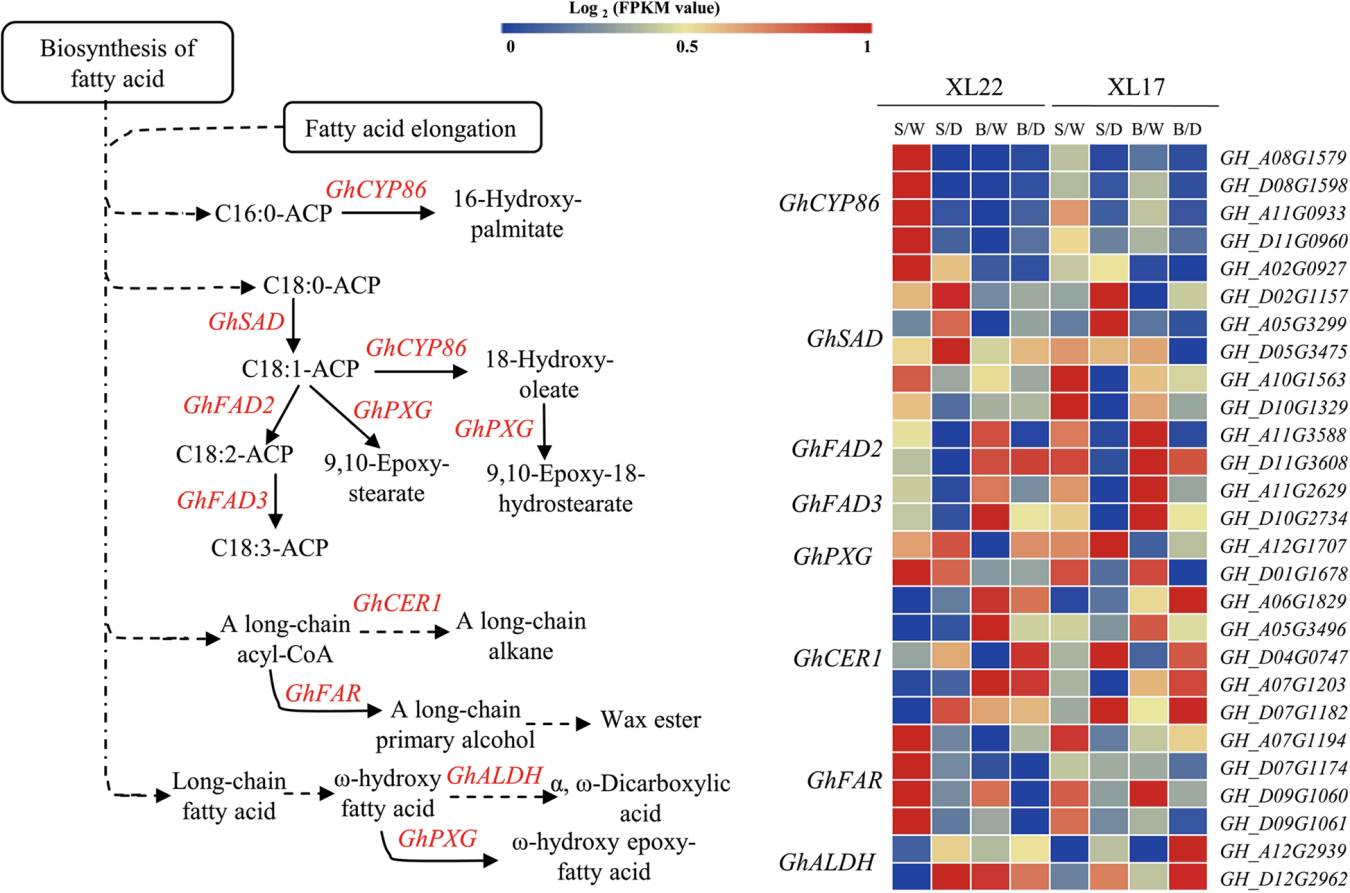
Expression profiles of the DEGs involved in biosynthesis of fatty acids, wax and cutinin XL22 and XL17. Left panel, the biosynthesis pathways of fatty acids, wax and cutin. Right panel, heatmap (the log2(FPKM value)) showing the expression patterns of the major genes of the biosynthesis pathways of fatty acids, wax and cutin. *GhCYP86*, Cytochrome P450 86A gene; *GhSAD*, stearoyl-ACP desaturase gene; *GhFAD2*, fatty acid desaturase 2 gene; *GhFAD*3, fatty acid desaturase 3 gene; *GhPXG*, peroxygenase gene; *GhCER*1, aldehyde decarbonylase gene; *GhFAR*, fatty acyl reductase gene; *GhALDH*, aldehyde dehydrogenase; C16:0-ACP, palmitic acid; C18:0-ACP, stearic acid; C18:1-ACP, oleic acid; C18:2-ACP, linoleic acid; C18:3-ACP, α-linolenic acid. S/W: well-watered plants at the seedling stage, S/D: water deficit plants at the seedling stage, B/W: well-watered plants at the bud stage, B/D: water deficit plants at the bud stage

Wax content was induced by drought stress in both XL22 and XL17, we therefore further investigated the DEGs involved in wax biosynthesis. *CER1*encodes an aldehyde decarbonylase that catalyzes biosynthesis of alkanes, a major step of wax production [16]. The cotton genome contains multiple *CER1* homologs. In the seedling stage, four *GhCER1* genes (*GH_A05G3496*, *GH_ D04G0747*, *GH_ A07G1203*, and *GH_ D07G1182*) were highly expressed in leaves. Of these genes, *GH_D07G1182* showed the highest expression level and was significantly induced by WD in both XL22 (increased by 72.5%) and XL17 (increased by 44.5%). At the bud stage, four *GhCER1* genes (*GH_A05G3496*, *GH_A06G1829*, *GH_A07G1203*, and *GH_D07G1182*) were highly expressed in leaves. Their expression levels were slightly decreased by WD in XL22, but the expression levels of *GH_A06G1829* and *GH_D07G1182* were significantly induced by WD in XL17. From the seedling stage to the bud stage, the *GhCER1* expression levels generally showed an increasing trend in both XL22 and XL17 (Fig. 7). Drought induced up-regulation of *GhCER1* in both XL22 and XL17 is consistent with the enhanced accumulation of leaf wax content under the WD conditions.

In the wax and cutin biosynthesis pathways, the ω-hydroxylation reaction is typically catalyzed by cytochrome P450 monooxygenases (CYP), and peroxygenase (PXG) catalyzes the hydroperoxide-dependent epoxidation of unsaturated fatty acids to produce epoxy-fatty acids. In the seedling stage, the expression level of *GhCYP86* genes (*GH_A11G0933*, *GH_D11G0960*, *GH_A08G1579*, and *GH_D08G1598*), which encode a very long chain fatty acid hydroxylase specifically involved in cutin biosynthesis, was significantly down-regulated by WD in both XL22 and XL17 (Fig. 7). However, in the bud stage, the expression levels of those *GhCYP86* genes were slightly induced by WD in XL22 and significantly repressed by WD in XL17. Of the *GhPXG* genes, a pair of homoeologs (*GH_A12G1707* and *GH_D01G1678*) was highly expressed in leaves of both XL22 and XL17 at the seedling stage, but displayed an opposite response to WD, with *GH_A12G1707* being significantly induced by WD. The transcript abundance of *GH_A12G1707* was also significantly induced by WD in both XL22 (> 4-folds) and XL17 (~ 2-folds) at the bud stage. *GhALDH* encodes an aldehyde dehydrogenase that catalyzes ω-oxo fatty acids to produce α, ω-dicarboxylic fatty acids. Four pairs of homoeologous *GhALDH* genes (*GH_A12G2939* and *GH_D12G2962*, *GH_A11G0436* and *GH_D11G0455*, *GH_A06G1679* and *GH_D06G1699*, *GH_A07G0707* and *GH_D07G0693*) exhibited high transcript abundance in leaves and their expression levels, especially *GH_A12G2939* and *GH_D12G2962*, were apparently induced by WD (Fig. 7). This was consistent with the significant induction of dicarboxylic acid by WD. Fatty acyl reductase (FAR) is a important enzyme involved in the synthesis of primary alcohols [44]. In *Gossypium hirsutum*, eight genes encoding FAR have been identified [33], and three of them, *GH_A07G1194* (*GhFAR3A.1*), *GH_D07G1174* (*GhFAR3A.2*), and *GH_D09G1060* (*GhFAR3D.2*) had high transcript abundance in leaves. Notably, the expression of those *GhFARs* was significant repressed by WD at both developmental stages, except *GH_A07G1194* at the bud stage (Fig. 7).

### Gene network analysis with WGCNA

**Fig. 8:**
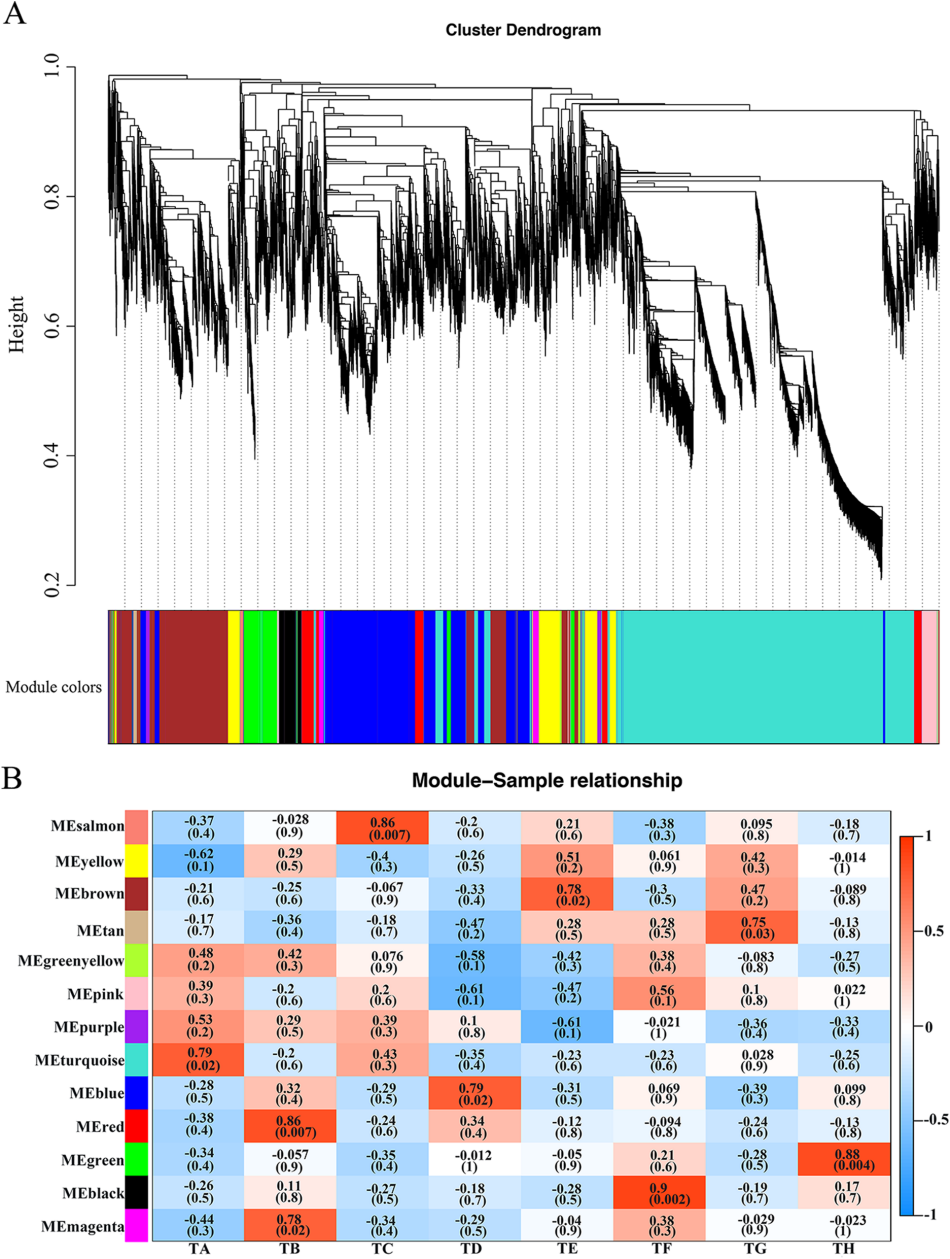
WGCNA of DEGs between the well-watered and WD plants of XL22 and XL17. **A** Hierarchical clustering tree of co-expression modules was analyzed by WGCNA and DEGs was divided into 13 modules that were defined by different color. **B** The correlation between modules and traits. Each row represents a module and each column corresponds to trait. The number in the rectangular box represents the correlation coefficient and corresponding *p*-value. Red represents the positive correlation and blue represents the negative correlation between the module and the trait. TA: well-watered XL22 at the seedling stage; TB: water deficit XL22 at the seedling stage; TC: well-watered XL17 at the seedling stage; TD: water deficit XL17 at the seedling stage; TE: well-watered XL22 at the bud; TF: water deficit XL22 at the bud stage; TG: well-watered XL17 at the bud stage; TH: water deficit XL17 at the bud stage

To identify the specific genes that were highly correlated with WD in cotton, the co-expression networks were generated by WGCNA using the all non-redundant DEGs. A total of 13 gene modules associated with the specific expression profiles of different samples were identified (Fig. 8A). Of the 13 modules, 5 (salmon, red, turquoise, black and green) were significantly associated with the two growth stages of XL22 and XL17 under the WW or WD conditions (Fig. 8B). The red module with 627 genes was highly associated with the seedling stage of XL22 under the WD conditions. The salmon with 54 genes was significantly associated with the seedling stage of XL17 under the WW conditions. The black (5440 genes) and green (690 genes) modules were significantly associated with the bud stage of XL22 and XL17, respectively, under the WD conditions (Fig. 8B). The 368 DEGs of the turquoise module were enriched with 23 GO terms, including microtubule binding, tubulin binding, cytoskeletal protein binding, movement of cell or subcellular component, and microtubule-based movement (Fig. S7A). The 5,440 DEGs of the black module were enriched with 43 GO terms, including oxidoreductase activity, dioxygenase activity, methionine adenosyltransferase activity, endopeptidase inhibitor activity, and peptidase inhibitor activity (Fig. S7B). KEGG analysis of the DEGs of the turquoise and black modules found that the turquoise module was enriched with genes involved in DNA replication (*P*_*adj*_ = 1.29 × 10^–6^, 31 genes) for the seedling stage of XL22 under the WW conditions (Fig. S7C), while the black module was enriched with genes involved in the linoleic acid metabolic pathway (*P*_*adj*_ = 5.78 × 10^–5^, 6 genes) for the bud stage of XL22 under the WD conditions (Fig. S7D).

### Co-expression gene networks and hub genes

**Fig. 9:**
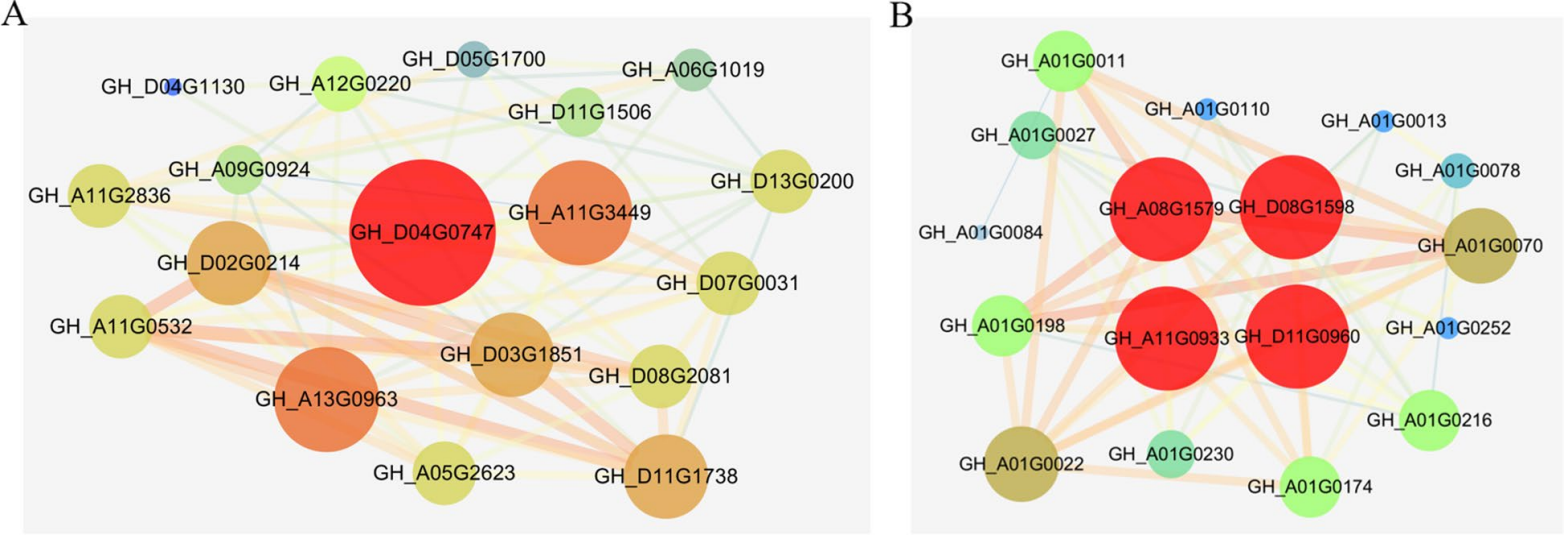
Co-expression network analysis. **A** Co-expression network analysis results of the black module. **B** Co-expression network analysis results of the turquoise module. Red circles represent the hub genes. Circles size and color represent the degree. Line size represents the weight

According to the different plant phenotypes and wax compositions and cutin monomers between XL22 and XL17 under WW and WD conditions, we further analyzed the co-expression networks of DEGs of the black and turquoise modules to identify the hub genes. Based on the criteria of eigengene-based connectivity (*K*_*ME*_) value ≥ 0.84 and high weight value ≥ 0.19, 18 genes were found to be co-expressed in the black module. One of the *GhCER1* genes, *GH_D04G0747*, was identified to be the hub genes of the black module (Fig. 9A). Based on the criteria of eigengene-based connectivity (*K*_*ME*_) value ≥ 0.95 and high weight value ≥ 0.14, 17 genes were found to be co-expressed in the turquoise module. Four *GhCYP86* genes (*GH_A08G1579*, *GH_D08G1598*, *GH_A11G0933*, and *GH_D11G0960*) encoding cytochrome P450 were identified to be the hub genes of the turquoise module (Fig. 9B). We found that these genes are related to the biosynthesis of unsaturated fatty acids, wax and cutin, and their expression might be sensitive to WD.

### Expression profiles of the genes involved in biosynthesis of unsaturated fatty acids

Plants could adapt to environmental stresses by changing the proportion of unsaturated fatty acids in their membrane lipids. Therefore, the effect of water deficiency on the expression levels of the key genes of the unsaturated fatty acid synthesis pathway was further analyzed. C18:0-ACP is desaturated by ^Δ9^-stearoyl-ACP desaturase (SAD) to form monounsaturated C18:1n-9-ACP. Six *GhSAD* genes (*GH_A10G1563*, *GH_D10G1329*, *GH_A05G3299*, *GH_D05G3475*, *GH_A02G0927*, and *GH_D02G1157*) identified in the cotton genome had high expression levels in leaves. Of those *GhSAD* genes, the expression levels of a pair of homoeologs (*GH_A10G1563* and *GH_D10G1329*) were much higher than that of their homologs, which means that this pair of *GhSAD* could take a leading role in C18:0-ACP desaturation in leaves. WD treatment reduced the abundance of *GH_A10G1563* and *GH_D10G1329* transcripts in leaves at the seedling stage of XL22 and XL17, whereas WD had no significant effect on the expression of the two genes in leaves at the bud stage. The major polyunsaturated fatty acid (PUFA) in cottonseed is synthesized by fatty acid desaturase 2 (*FAD2*, C18:1n-9 to C18:2n-6 desaturation) and polyunsaturated linolenic acid (C18:3n-3) is desaturated from C18:2n-6 by fatty acid desaturase 3 (*FAD3*). Four pairs of homoeologous *GhFAD2* with different expression patterns have been identified in the cotton genome [37]. The homoeologous pair of *GH_A11G3588* and *GH_D11G3608* were highly expressed in cotton leaves, indicating that they might be the major contributors in catalyzing C18:2n-6 biosynthesis in cotton leaves. Under the WD conditions, the expression levels of *GH_ A11G3588* and *GH_D11G3608* decreased significantly in leaves of XL17 and XL22 at the seedling stage, resulting in a lower accumulation of C18:2n-6 in leaves. In the WW plants, the expression levels of *GH_ A11G3588* and *GH_D11G3608* in both XL17 and XL22 increased slightly from the seedling stage to the bud stage, consistent with a higher accumulation of C18:2n-6 in leaves of both varieties at the bud stage. Compared with the WW plants, the WD-treated plants had a significantly decreased expression level of *GH_A11G3588* and a similar level of *GH_D11G3608* at the bud stage. Consequently, the accumulation of C18:2n-6 in leaves under the WD conditions was lower than that under the WW conditions at the bud stage. In cotton, FAD3 is mainly encoded by a pair of homoeologous *GhFAD3* genes (*GH_A10G2629* and *GH_D10G2734*). Their expression level in leaves was significantly repressed by WD at the bud stage, consistent with decreased accumulation of C18:3n-6 in leaves of XL22 and XL17 under the WD conditions. In *Gossypium*, the low temperature could induce an increase in the mRNA levels of *GhFAD2* to produce more C18:2n-6 [45]. Different from low temperature stress, our results showed that under WD condition, the significant decrease of PUFA was consistent with those reported under salt stress [46–48].

## Discussion

Drought is increasingly becoming one of the serious abiotic stresses inhibiting crop productivity. Drought stress significantly affects plant growth and development by reducing plant height and leaf area [49]. Understanding the physiological and molecular responses of plants induced by WD is the key to find solutions for mitigating the effect of drought stress on crop productivity. In this study, using two Upland cotton varieties tolerant (XL22) or sensitive (XL17) to drought stress, we compared their stoma-dependent (stomal density and aperture) and stoma-independent (leaf wax and cutin) responses to WD, and did comparative transcriptomic analysis to identify drought responsive genes and networks.

Under the WW conditions, there was no significant difference in stomatal density per unit of leaf area, leaf cuticular wax content, and RWC between the drought tolerant XL22 and the drought sensitive XL17, although XL17 seemed to consume slightly more water daily than XL22 during the period from 30 to 50 days after seedling emergence. WD stress increased stomata density per unit of leaf area and reduced stomatal aperture in both XL22 and XL17. The drought-induced increase in stomatal density per unit of leaf area had also been observed in *Triticum aestivum* [50]. In contrast, reduced stomatal density was observed in *Populus balsamifera* under WD condition [51]. Xu and Zhou reported an increase in the stomatal density on leaves of *Leymus chinensis* under moderate drought stress, while stomatal density tended to decrease under more severe drought stress conditions [52]. Under drought conditions, there was no significant change in stomatal density of *Arachis hypogaea* [53]. Taking into account the decrease of leaf area under WD conditions, compared with that of the controls, the total stomatal density of XL22 and XL17 leaves decreased respectively by 32% and 40% in our study, although the stomatal density per unit leaf area increased. The negative relationship between stomatal density and aperture has been previously documented in other plants under WD conditions [50, 54, 55]. The balance between stomatal density and size is coordinated that is related to the limitation of the leaf area allocated to stomata [56]. Bi et al. [50] found that the response of stomatal density to drought varies with different varieties and the difference in stomatal density under drought could not explain the difference of water loss rate among varieties, and thought that the cuticle composition is important for water loss, rather than a simple correlation with stomatal density under WD conditions. In our study, WD stress increased stomata density per unit leaf area and decreased stomatal aperture in both XL22 and XL17, also suggesting the importance of stomata in regulation of drought stress.

Previous studies have shown that WD-induced increase of cuticle wax produces a cuticle layer with poor water permeability to limit transpiration and delay the occurrence of cell dehydration stress [10, 57, 58]. It has also been reported that WD increases the amounts of nearly all leaf cutin monomers [1]. We also found that WD stress had a significant effect on the accumulation of cuticular wax and cutin in cotton leaves. Accumulation of two of the major leaf wax components, the ultra-long chain C29 and C31 alkanes, and one of the major cutin monomers, palmitic acid (C16:0), were significantly induced by WD stress in both XL22 and XL17, whereas accumulation of one of the major cutin monomers, linolenic acid (C18:3n-3) was significantly repressed by WD stress in both XL22 and XL17. Two cutin monomers, hexadecane-1,16-dioic acid (C16:0 DCA) and octadecanedioic acid (C18:0 DCA), were induced by WD stress in both XL22 and XL17, with a more significant induction observed in the drought sensitive XL17. These results suggest that drought stress induced both stoma-dependent and stoma-independent responses in drought tolerant or sensitive cotton varieties and the role of C16:0 DCA and C18:0 DCA in stoma-independent drought tolerance is yet to be further investigated.

Leaf epidermal wax is the protective layer covering the outermost layer of plants. It can effectively prevent the non-stomatal loss of water in plants and reduce transpiration, and plays an important role in maintaining leaf water content [1, 33]. After water deficiency, the increases of leaf wax amount in XL22 and XL17 indicate that their wax biosynthesis pathways were enhanced by drought stress. Similar results have been previously reported in other plants [10, 11, 13, 58–61]. Our results also showed that alkanes are the main waxy components in cotton leaves and the most notable change in the wax constituent profile in WD cotton leaves was the increase of alkanes, consistent with the results reported in *Arabidopsis* [1]. *CER1* plays an important role in alkane synthesis [15, 62]. Overexpression of *CER1* in plants showed decreased cuticle permeability and reduced sensitivity to soil WD [16]. In this study, *GhCER1* was identified to be the hub genes of the black module. Transcriptome analysis showed that water deficiency induced the expression of *GhCER1*, which might contribute to the increase of the total content of alkanes, the major components of leaf wax, in leaves of the WD cotton plants.

Fatty alcohols are important components of aliphatics and have a crucial role in cuticle permeability and biosynthesis of wax, suberin and cutin [37, 63]. Studies presented here demonstrated that the relative content of fatty alcohols decreased significantly under the WD conditions. RNA-seq analysis showed that genes encoding the long chain fatty acid hydroxylase, such as *GhCYP86* and *GhFAR*, were less abundant in cotton leaves experienced WD. *AtFAR3/CER4* encodes FAR involved in the production of long chain primary fatty alcohols of *Arabidopsis* leaf cuticular waxes [64]. The expression of several genes including *AtFAR3* could be activated by *MYB94* transcription factor and the amounts of hydroxy fatty acids increased by 39% in MYB94-overexpressing line relative to the WT [65]. Under WD conditions, compared to that in WT leaves, cuticular transpiration occurred more slowly in MYB94-overexpressing line leaves due to the increase in cuticular wax amount [65]. Silencing *GhFAR* in cotton leads to increased susceptibility to drought stress [33] and complete elimination of fatty alcohols is lethal to plants [63]. During biosynthesis of the cutin monomers, the ω-hydroxylation reaction can be catalyzed by cytochrome P450 monooxygenases, e.g. *CYP86* [22, 37]. It had also been reported that the mutants in P450-genes encoding fatty acid ω-hydroxylases presumably involved in the pathway to α, ω-diacids, show increased permeability to water vapor [22] or become permeable for the lipid boundary of the outer wall of epidermal cells [21]. Consistent with the gene expression data, the total contents of hydroxyacids notably decreased in response to WW conditions. Interestingly, under the WD conditions, a significant expression difference of the *GhCYP86* genes (*GH_A11G0933*, *GH_D11G0960*, *GH_A08G1579*, and *GH_D08G1598*) was observed between XL22 and XL17 at both the seedling and the bud stages. Decrease of the *GhCYP86* (*GH_A11G0933*, *GH_D11G0960*, *GH_A08G1579*, and *GH_D08G1598*) transcription levels caused by water shortage was much greater in XL17 than in XL22. *GH_A11G0933*, *GH_D11G0960*, *GH_A08G1579*, and *GH_D08G1598* were also identified to be the hub genes of the turquoise module. Consistent with the transcriptional abundance of *GhCYP86*, a more significant decrease of the fatty alcohol content was observed in drought sensitive XL17 than in drought tolerant XL22. Whether the different expression changes of these genes in XL22 and XL17 might contribute to the drought sensitivity of the two varieties is an open question.

## Conclusions

In conclusion, WD stress changes stomatal density and aperture as well as accumulation of wax and cutin in leaves in both drought tolerant and sensitive cotton varieties. WD-induced changes of certain wax components and cutin monomers specific to XL22 or XL17 were observed, implying their potential role in drought tolerance or sensitivity, which is worth of further investigation. Transcriptomic analysis identified DEGs between WW and WD as well as between XL22 and XL17, and hub genes and their associated gene networks related to response to drought stress. *GhCYP86* (*GH_A11G0933*, *GH_D11G0960*, *GH_A08G1579*, and *GH_D08G1598*) encoding long chain fatty acid hydroxylase are particularly of interest, as they showed consistent differential expression between XL22 and XL17 under the WW and WD conditions, implying their potential contribution to the difference of drought tolerance of the two cotton varieties.

## Supplementary Information

**Additional file 1: Table S1.** Yield in field trials of XL22 and XL17 under the WW and WD conditions.

**Additional file 2: Table S2.** Primers used in qRT-PCR analysis.

**Additional file 3: Table S3.** Transcriptome sequencing data quality and genome mapping.

**Additional file 4: Table S4.** Analysis of GO enrichment for 15,303 common DEGs.

**Additional file 5: Table S5.** Analysis of GO enrichment from the seedling stage and bud stage of XL22.

**Additional file 6: Table S6.** Analysis of KEGG enrichment of the DEGs.

**Additional file 7:** **Fig. S1.** Consumed water analysis of XL22 and XL17 under the WW and WD conditions. A. Plant phenotyping of XL22 and XL17 at bud stage under the WW and WD conditions. B. Consumed water content of XL22 and XL17 under the WW and WD conditions. WW, well-watered; WD, water deficit. The scales bars are indicated with white lines. Error bars are standard errors. Values represent the means ± SE, n = 3. Different letters above the bars indicate statistically different from each other as determined by the Student’s t test: p < 0.05. **Fig. S2.** SEM observation of the leaves surface wax of XL22 and XL17 under the WW and WD conditions. a and b. XL22 leaf at the seedling stage under WW and WD condition, respectively. c and d. XL22 leaf at the bud stage under WW and WD condition, respectively. e and f. XL17 leaf at the seedling stage under WW and WD condition, respectively. g and h. XL17 leaf at the bud stage under WW and WD condition, respectively. i, j, k and l, the adaxial surface of XL22 leaf corresponds to a, b, c and d. m, n, o and p, the adaxial surface of XL17 leaf corresponds to e, f, g and h. The waxy crystals as indicated by the arrows. WW, well-watered; WD, water deficit. SS-22, XL22 leaves at seedling stage; SS-17, XL17 leaves at seedling stage; BS-22, XL22 leaves at bud stage; BS-17, XL17 leaves at bud stage. **Fig. S3.** Total wax content of cotton leaves of XL22 and XL17 under WW and WD conditions. WW, well-watered; WD, water deficit. SS, the seedling stage; BS, the bud stage. Error bars are standard errors. Values represent the means ± SE, n = 3. Different letters above the bars indicate statistically different from each other as determined by the Student’s t test: p < 0.05. **Fig. S4.** Heatmap showing the relative expression levels of the 9 selected genes in the two cotton varieties determined by RNA-seq analysis (A) and qRT-PCR (B). Of the selected genes, 4 genes are involved in fatty acid synthesis and 5 genes are related to wax and cutin biosynthesis. The enzymes encoded by these genes are: Very-long-chain enoyl-CoA reductase (GH_D10G2517), Very-long-chain 3-oxoacyl-CoA reductase 1 (GH_D03G1424), 3-oxoacyl-acyl-carrier-protein synthase II (GH_A13G2186), Stearoyl-acyl-carrier-protein 9-desaturase (GH_D10G1329), Delta(12)-fatty-acid desaturase (GH_A11G3588), Probable peroxygenase 4 (GH_A12G1707), Cytochrome P450 86A22 (GH_A08G2095), Very-long-chain -3-hydroxyacyl-CoA dehydratase (GH_D11G2259), Very-long-chain-3-hydroxyacyl-CoA dehydratase 2 (GH_A03G0493). S/W: well-watered plants at the seedling stage, S/D: water deficit plants at the seedling stage, B/W: well-watered plants at the bud stage, B/D: water deficit plants at the bud stage. GhUBI was used as a reference gene. All qRT–PCR reactions were performed in triplicate. **Fig. S5.** KEGG analysis of DEGs at the seedling stage of XL22 and XL17. A. KEGG categories of DEGs at the seedling stage of XL22. B. KEGG categories of DEGs at the seedling stage of XL17. **Fig. S6.** Heatmap comparison of DEGs associated with two cotton varieties. A. Venn diagram showing the different KEGGs at the two stages of XL22 and XL17. B and C show the DEGs at the seedling stage of XL22. D and E show the DEGs at the seedling stage of XL17. Red represents high expression, and blue represents low expression. Each row represents a DEG. S/W-22: well-watered XL22 at the seedling stage; S/D-22: water deficit XL22 at the seedling stage; S/W-17: well-watered XL17 at the seedling stage; S/D-17: water deficit XL17 at the seedling stage; B/W-22: well-watered XL22 at the bud stage; B/D-22: water deficit XL22 at the bud stage; B/W-17: well-watered XL17 at the bud stage; B/D-17: water deficit XL17 at the bud stage.** Fig. S7.** GO analysis and KEGG pathways of the black module and turquoise module genes. A. Analysis of GO enrichment of the black module genes, B. Analysis of GO enrichment of the turquoise module genes, C. KEGG categories of DEGs of the black module, D. KEGG categories of DEGs of the turquoise module. The horizontal axis represents rich factor, the vertical axis represents statistics of the enriched pathways. Circle size represents the number of genes. Red represents high KEGG enrichment, and blue represents low KEGG enrichment.

## Data Availability

The datasets presented in this study can be found in online repositories. The names of the repository/repositories and accession number(s) can be found below: https://www.ncbi.nlm.nih.gov/bioproject/;PRJNA776142.
