# Supplementary material for: Impact of water deficiency on leaf cuticle lipids and gene expression networks in cotton (Gossypium hirsutum L.)
Source: BMC Plant Biol. 2022 Aug 17;22:404. doi: 10.1186/s12870-022-03788-2 (PMC9382817; doi:10.1186/s12870-022-03788-2)
Supplement: Supplementary file 1 — Additional file 1: Table S1. Yield in field trials of XL22 and XL17 under the WW and WD conditions. [file 12870_2022_3788_MOESM1_ESM.docx]

Table S1 Yield in field trials of XL22 and XL17 under the WW and WD conditions

| Cultivars | Treatment | Plant density  (10^4^/hm^2^) | Boll number  (per plant) | Single boll weight (g) | Lint percent  (%) | Seed cotton yield  (kg/hm^2^) |
| --- | --- | --- | --- | --- | --- | --- |
| XL17 | WW | 19.9 ± 0.4 a | 5.1 ± 0.1 a | 5.19 ± 0.10 a | 42.8 ± 0.6 a | 5 257 ± 218 a |
|  | WD | 19.9 ± 0.5 a | 3.3 ± 0.1 b | 4.71 ± 0.08 b | 41.1 ± 0.4 b | 3 084 ± 116 b |
| XL22 | WW | 20.1 ± 0.2 a | 5.2 ± 0.1 a | 5.25 ± 0.09 a | 43.0 ± 0.5 a | 5 514 ± 189 a |
|  | WD | 20.1 ± 0.5 a | 3.6 ± 0.2 b | 5.04 ± 0.08 a | 41.7 ± 0.3 b | 3 628 ± 111 b |

WW, well-watered; WD, water deficit. Error bars are standard errors. Values represent the means ± SE, n = 3. Different lowercase letters over columns indicate statistically different (*p* < 0.05) according to Duncan’s multiple range tests.
