# Supplementary material for: Impact of water deficiency on leaf cuticle lipids and gene expression networks in cotton (Gossypium hirsutum L.)
Source: BMC Plant Biol. 2022 Aug 17;22:404. doi: 10.1186/s12870-022-03788-2 (PMC9382817; doi:10.1186/s12870-022-03788-2)
Supplement: Supplementary file 2 — Additional file 2: Table S2. Primers used in qRT-PCR analysis. [file 12870_2022_3788_MOESM2_ESM.docx]

**Table S2 Primers used in qRT-PCR analysis**

| Gene ID | Predicted coding protein | Primer sequence (5’-3’) | Product length (bp) |
| --- | --- | --- | --- |
| *GH_D10G2517* | Very-long-chain enoyl-CoA reductase | F: ATCGCTACACAGACCGTTGC  R: ACCGTCGAATAGCTTCTTCAGT | 108 |
| *GH_D03G1424* | Very-long-chain 3-oxoacyl-CoA reductase 1 | F: CTACTGGCCCCATTCCATCC  R: CCCCTCTTCCGAATGCCAAG | 102 |
| *GH_A13G2186* | 3-oxoacyl-acyl-carrier-protein synthase II, chloroplastic | F: CCGATGCAGCGATTATACCCA  R: GATTAGCATCCCAAGGGCGT | 108 |
| *GH_D10G1329* | Stearoyl-acyl-carrier-protein 9-desaturase, chloroplastic | F: CTTTGGAGGACTGGGCTGAG  R: CGTGAAATCCATCTGATGCAGG | 108 |
| *GH_A11G3588* | Delta(12)-fatty-acid desaturase FAD2 | F: CGCAATGGAAGCAACTAAGGC  R: CTCTGCTCACCTTCGTCTGG | 135 |
| *GH_A12G1707* | Probable peroxygenase 4 | F: AATTCGCTCGCACTCATTCG  R: GTCCAGCTAGCAACCCATCC | 106 |
| *GH_A08G2095* | Cytochrome P450 86A22 | F: GTACTGTTACGCCACCGTCT  R: GGTGCACGTCCATCACTAGA | 103 |
| *GH_D11G2259* | Very-long-chain (3R)-3-hydroxyacyl-CoA dehydratase PASTICCINO 2 | F: GTTCTTCTTTGCAGGTTTCAGGT  R: AAAACCTCCATAACGGCAGC | 131 |
| *GH_A03G0493* | Very-long-chain (3R)-3-hydroxyacyl-CoA  dehydratase 2 | F: GCCGGATTCACGAGGTACAG  R: GTAGGTGAGCCAAGACGGAC | 131 |

Gene IDs and protein annotations were based on Hu et al. (2019).
