# Supplementary material for: Impact of water deficiency on leaf cuticle lipids and gene expression networks in cotton (Gossypium hirsutum L.)
Source: BMC Plant Biol. 2022 Aug 17;22:404. doi: 10.1186/s12870-022-03788-2 (PMC9382817; doi:10.1186/s12870-022-03788-2)
Supplement: Supplementary file 7 — Additional file 7: Fig. S1. Consumed water analysis of XL22 and XL17 under the WW and WD conditions. A. Plant phenotyping of XL22 and XL17 at bud stage under the WW and WD conditions. B. Consumed water content of XL22 and XL17 under the WW and WD conditions. WW, well-watered; WD, water deficit. The scales bars are indicated with white lines. Error bars are standard errors. Values represent the means ± SE, n = 3. Different letters above the bars indicate statistically different from each other as determined by the Student’s t test: p < 0.05. Fig. S2. SEM observation of the leaves surface wax of XL22 and XL17 under the WW and WD conditions. a and b. XL22 leaf at the seedling stage under WW and WD condition, respectively. c and d. XL22 leaf at the bud stage under WW and WD condition, respectively. e and f. XL17 leaf at the seedling stage under WW and WD condition, respectively. g and h. XL17 leaf at the bud stage under WW and WD condition, respectively. i, j, k and l, the adaxial surface of XL22 leaf corresponds to a, b, c and d. m, n, o and p, the adaxial surface of XL17 leaf corresponds to e, f, g and h. The waxy crystals as indicated by the arrows. WW, well-watered; WD, water deficit. SS-22, XL22 leaves at seedling stage; SS-17, XL17 leaves at seedling stage; BS-22, XL22 leaves at bud stage; BS-17, XL17 leaves at bud stage. Fig. S3. Total wax content of cotton leaves of XL22 and XL17 under WW and WD conditions. WW, well-watered; WD, water deficit. SS, the seedling stage; BS, the bud stage. Error bars are standard errors. Values represent the means ± SE, n = 3. Different letters above the bars indicate statistically different from each other as determined by the Student’s t test: p < 0.05. Fig. S4. Heatmap showing the relative expression levels of the 9 selected genes in the two cotton varieties determined by RNA-seq analysis (A) and qRT-PCR (B). Of the selected genes, 4 genes are involved in fatty acid synthesis and 5 genes are related to wax and cutin biosynthesis. [file 12870_2022_3788_MOESM7_ESM.doc]

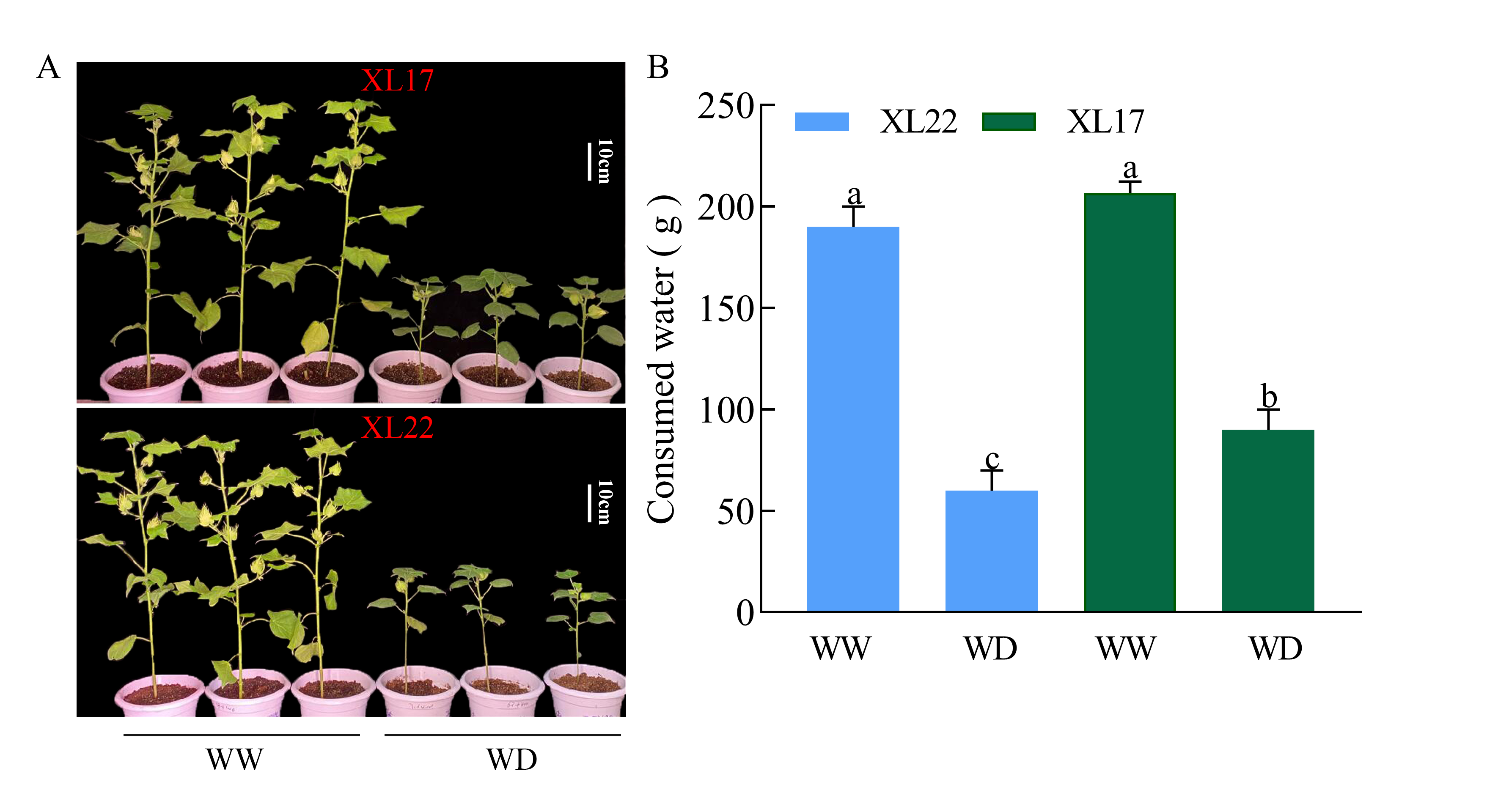


**Fig. S1 Consumed water analysis of XL22 and XL17 under the WW and WD conditions**

**A.** Plant phenotyping of XL22 and XL17 at bud stage under the WW and WD conditions. **B.** Consumed water content of XL22 and XL17 under the WW and WD conditions. WW, well-watered; WD, water deficit. The scales bars are indicated with white lines. Error bars are standard errors. Values represent the means ± SE, n = 3. Different letters above the bars indicate statistically different from each other as determined by the Student’s *t* test: *p* < 0.05.


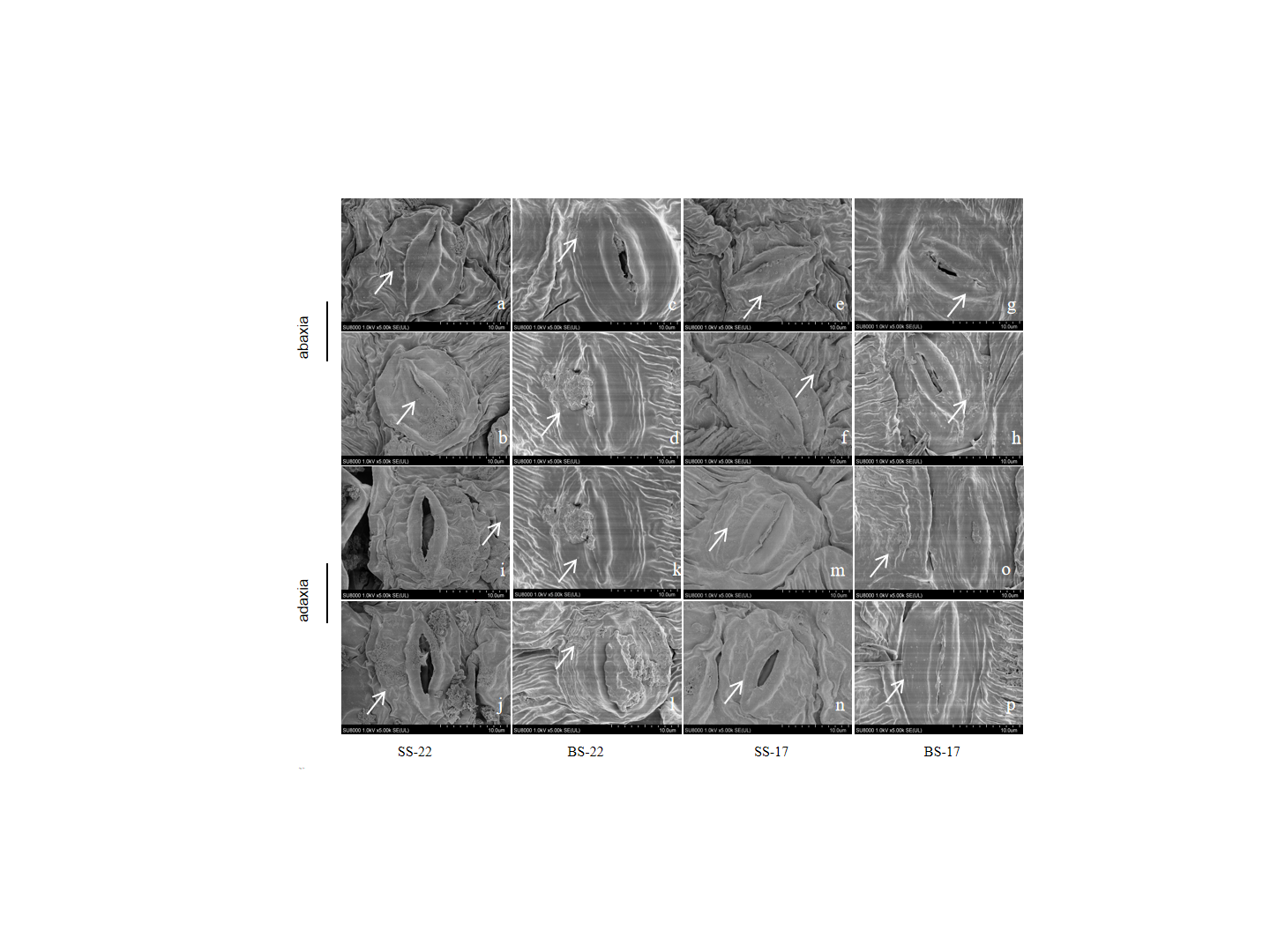


**Fig. S2 SEM observation of the leaves surface wax of XL22 and XL17 under the WW and WD conditions**

**a** and **b**. XL22 leaf at the seedling stage under WW and WD condition, respectively. **c** and **d**. XL22 leaf at the bud stage under WW and WD condition, respectively. **e** and **f**. XL17 leaf at the seedling stage under WW and WD condition, respectively. **g** and **h**. XL17 leaf at the bud stage under WW and WD condition, respectively. **i**, **j**, **k** and **l**, the adaxial surface of XL22 leaf corresponds to **a**, **b**, **c** and **d**. **m**, **n**, **o** and **p**, the adaxial surface of XL17 leaf corresponds to **e**, **f**, **g** and **h**. The waxy crystals as indicated by the arrows. WW, well-watered; WD, water deficit. SS-22, XL22 leaves at seedling stage; SS-17, XL17 leaves at seedling stage; BS-22, XL22 leaves at bud stage; BS-17, XL17 leaves at bud stage.


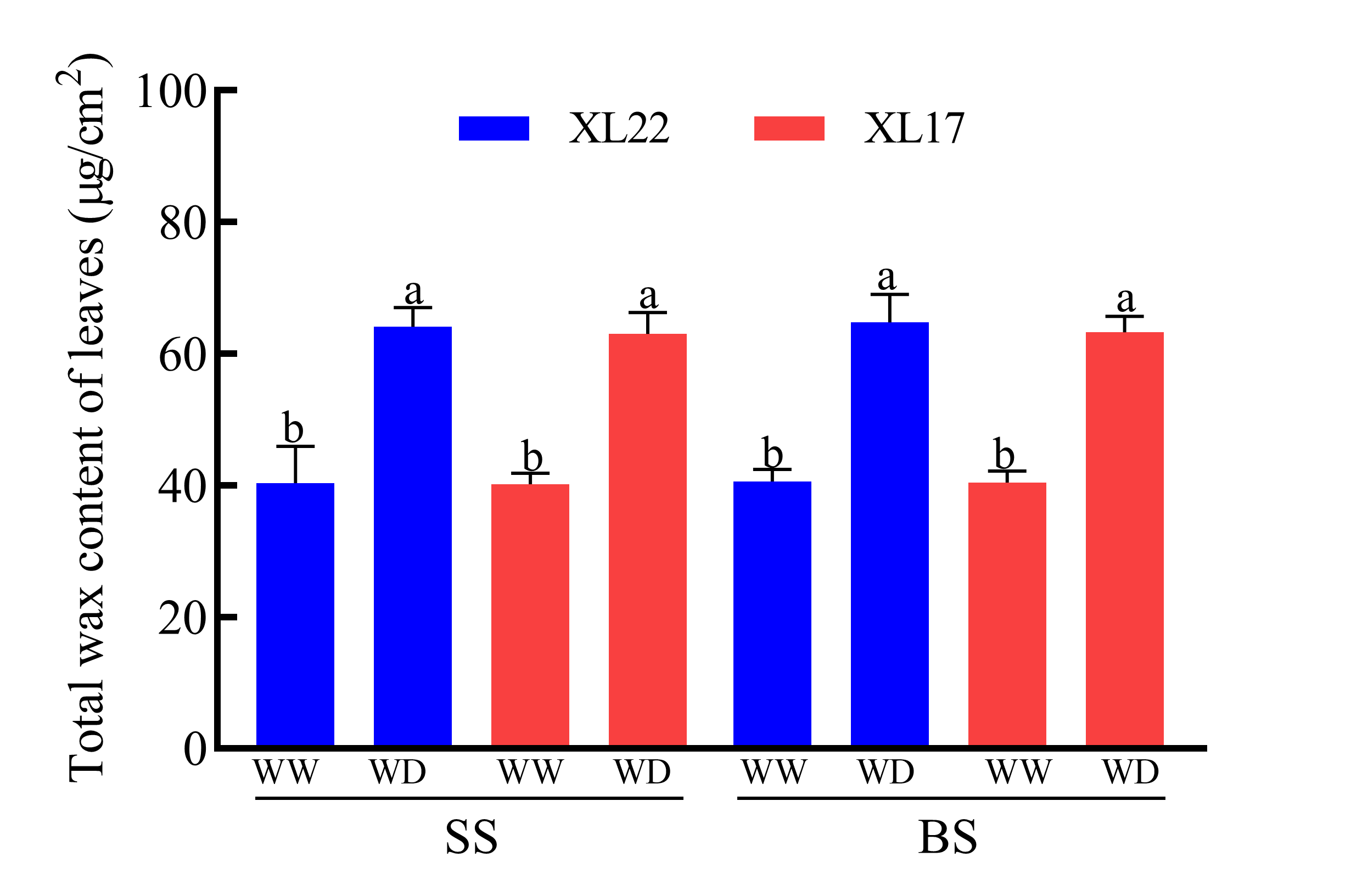


**Fig. S3 Total wax content of cotton leaves of XL22 and XL17 under WW and WD conditions**

WW, well-watered; WD, water deficit. SS, the seedling stage; BS, the bud stage. Error bars are standard errors. Values represent the means ± SE, n = 3. Different letters above the bars indicate statistically different from each other as determined by the Student’s *t* test: *p* < 0.05.


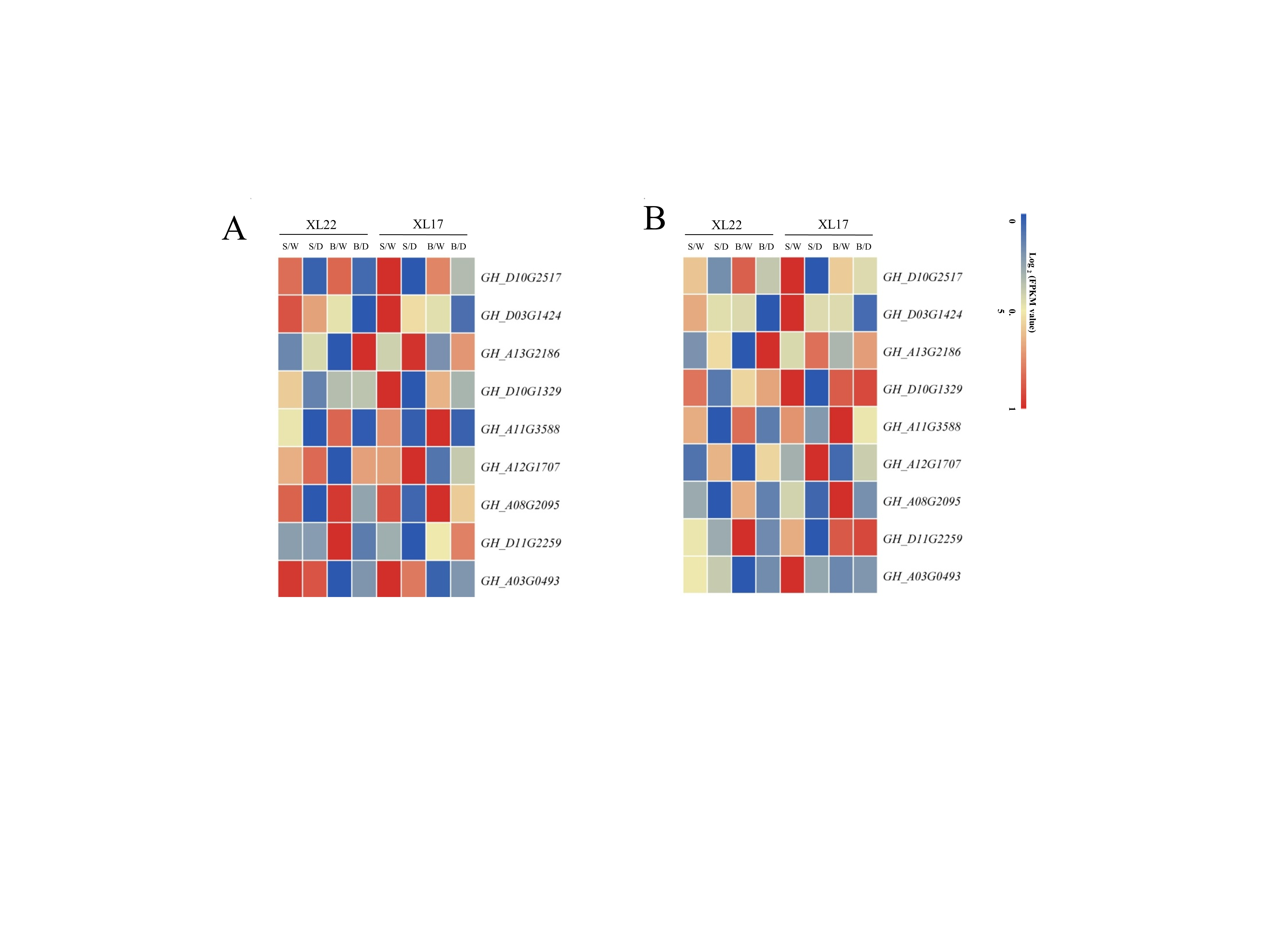


**Fig. S4 Heatmap showing the relative expression levels of the 9 selected genes in the two cotton varieties determined by RNA-seq analysis (A) and qRT-PCR (B)**

Of the selected genes, 4 genes are involved in fatty acid synthesis and 5 genes are related to wax and cutin biosynthesis. The enzymes encoded by these genes are: Very-long-chain enoyl-CoA reductase (*GH_D10G2517*), Very-long-chain 3-oxoacyl-CoA reductase 1 (*GH_D03G1424*), 3-oxoacyl-acyl-carrier-protein synthase II (*GH_A13G2186*), Stearoyl-acyl-carrier-protein 9-desaturase (*GH_D10G1329*), Delta(12)-fatty-acid desaturase (*GH_A11G3588*), Probable peroxygenase 4 (*GH_A12G1707*), Cytochrome P450 86A22 (*GH_A08G2095*), Very-long-chain -3-hydroxyacyl-CoA dehydratase (*GH_D11G2259*), Very-long-chain-3-hydroxyacyl-CoA dehydratase 2 (*GH_A03G0493*). S/W: well-watered plants at the seedling stage, S/D: water deficit plants at the seedling stage, B/W: well-watered plants at the bud stage, B/D: water deficit plants at the bud stage. *GhUBI* was used as a reference gene. All qRT–PCR reactions were performed in triplicate.


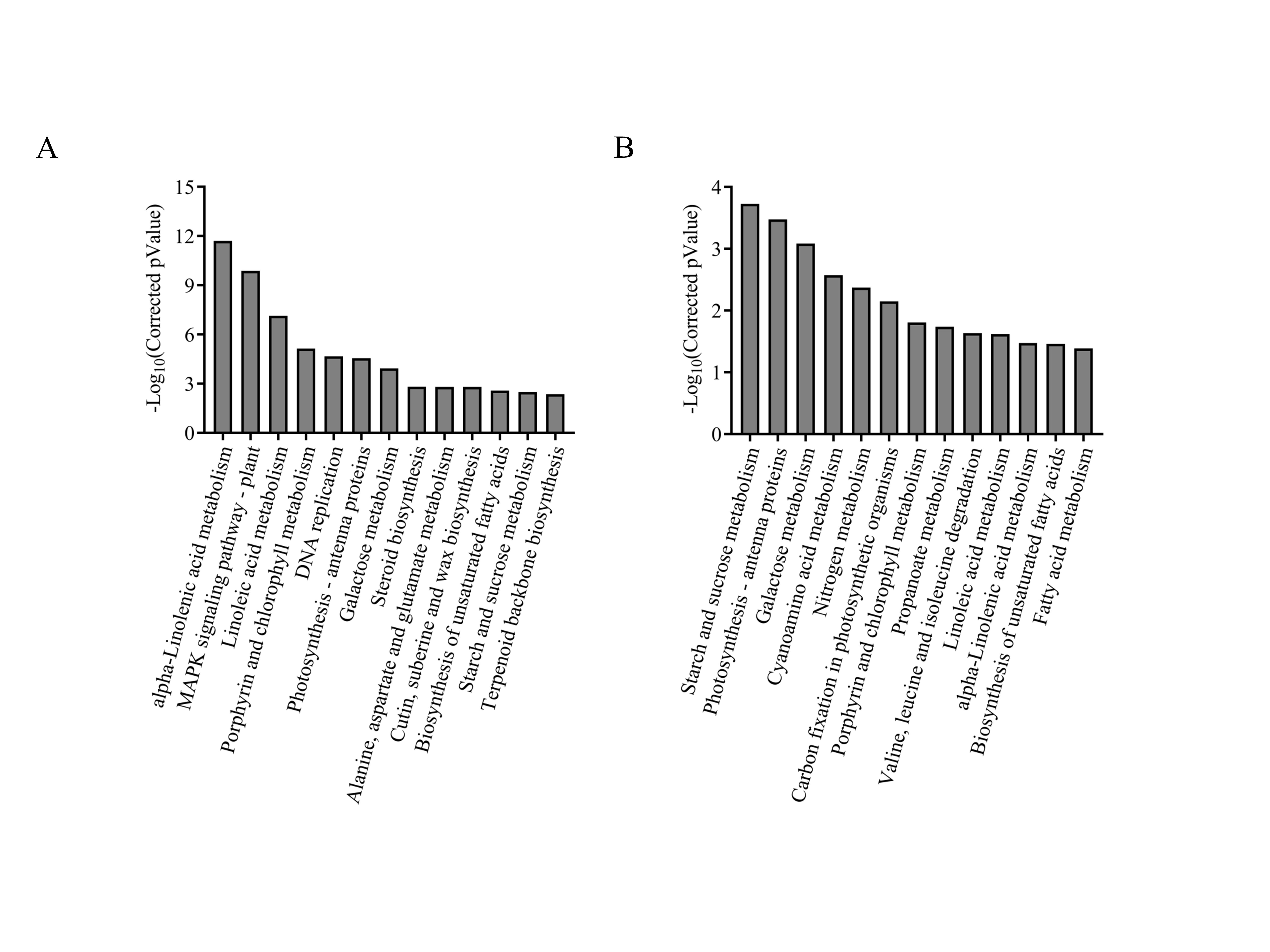


**Fig. S5 KEGG analysis of DEGs at the seedling stage of XL22 and XL17**

**A.** KEGG categories of DEGs at the seedling stage of XL22. **B.** KEGG categories of DEGs at the seedling stage of XL17.


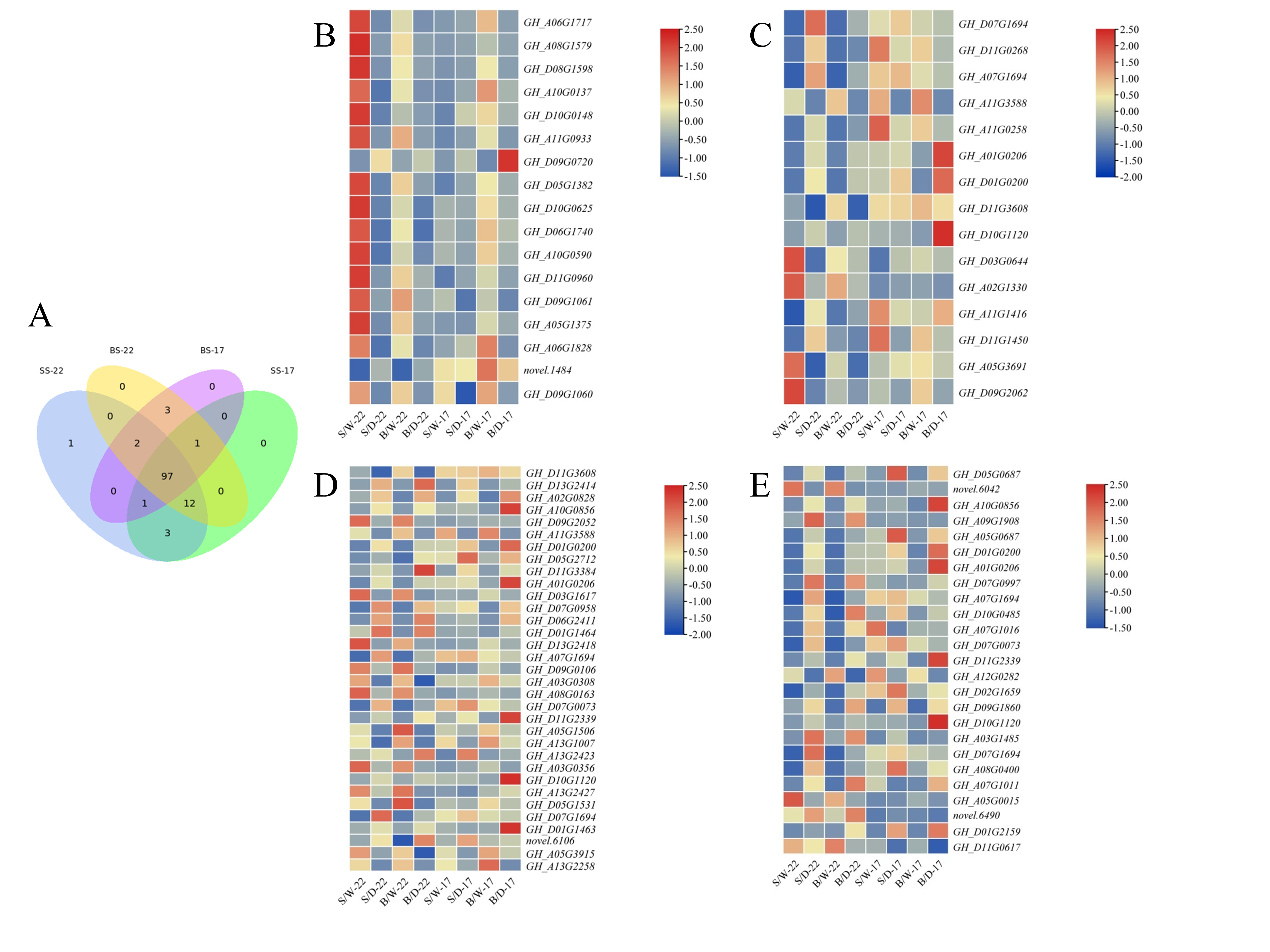


**Fig. S6 Heatmap comparison of DEGs associated with two cotton varieties**

**A.** Venn diagram showing the different KEGGs at the two stages of XL22 and XL17. **B** and **C** show the DEGs at the seedling stage of XL22. **D** and **E** show the DEGs at the seedling stage of XL17. Red represents high expression, and blue represents low expression. Each row represents a DEG. S/W-22: well-watered XL22 at the seedling stage; S/D-22: water deficit XL22 at the seedling stage; S/W-17: well-watered XL17 at the seedling stage; S/D-17: water deficit XL17 at the seedling stage; B/W-22: well-watered XL22 at the bud stage; B/D-22: water deficit XL22 at the bud stage; B/W-17: well-watered XL17 at the bud stage; B/D-17: water deficit XL17 at the bud stage.


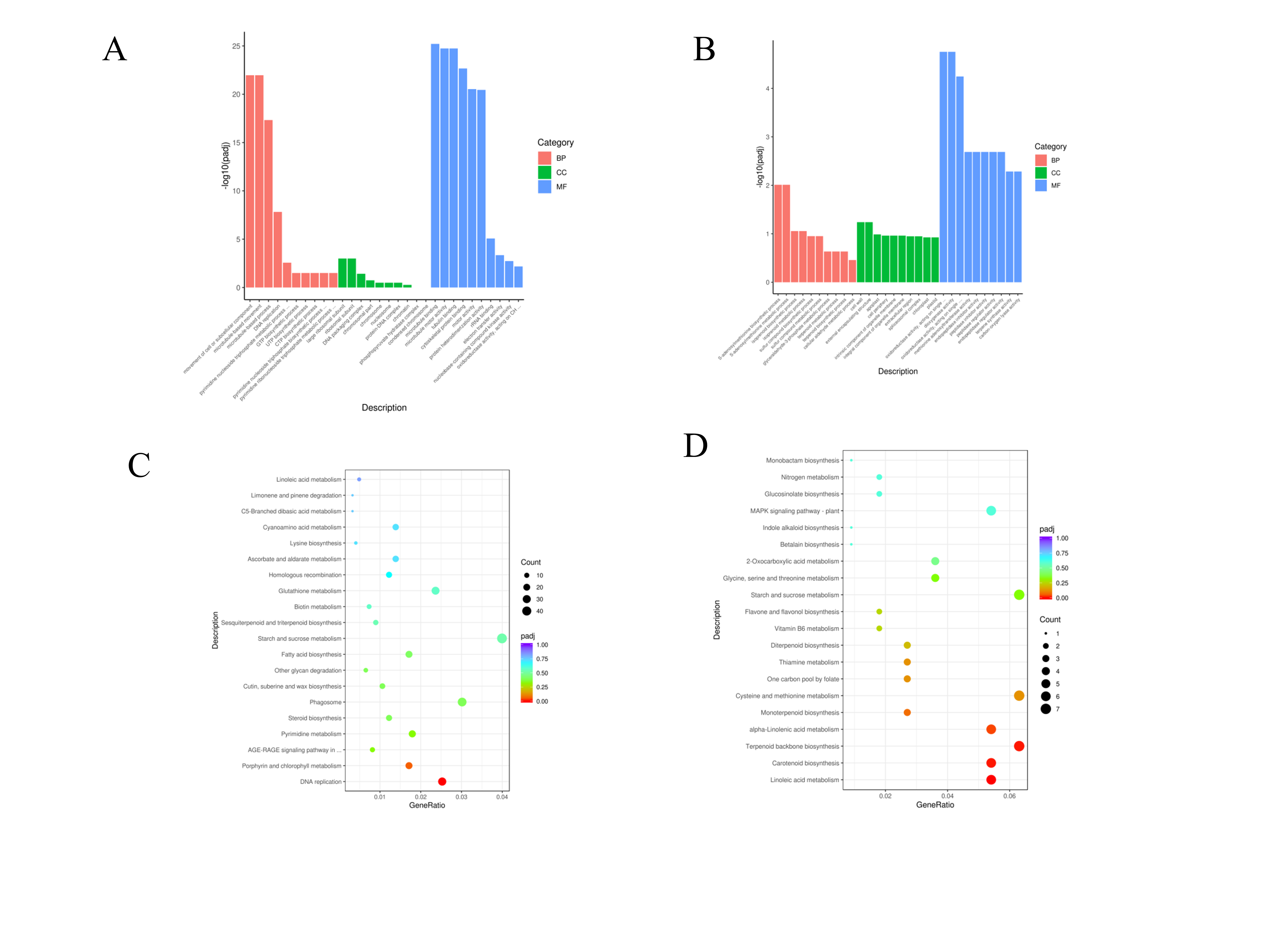


**Fig. S7 GO analysis and KEGG pathways of the black module and turquoise module genes**

**A.** Analysis of GO enrichment of the black module genes, **B.** Analysis of GO enrichment of the turquoise module genes, **C.** KEGG categories of DEGs of the black module, **D.** KEGG categories of DEGs of the turquoise module. The horizontal axis represents rich factor, the vertical axis represents statistics of the enriched pathways. Circle size represents the number of genes. Red represents high KEGG enrichment, and blue represents low KEGG enrichment.
